# ATP and adenosine—Two players in the control of seizures and epilepsy development

**DOI:** 10.1016/j.pneurobio.2021.102105

**Published:** 2021-06-16

**Authors:** Edward Beamer, Manvitha Kuchukulla, Detlev Boison, Tobias Engel

**Affiliations:** a Department of Physiology and Medical Physics, Royal College of Surgeons in Ireland, University of Medicine and Health Sciences, Dublin D02 YN77, Ireland; b Centre for Bioscience, Manchester Metropolitan University, John Dalton Building, All Saints Campus, Manchester M15 6BH, UK; c Department of Neurosurgery, Robert Wood Johnson & New Jersey Medical Schools, Rutgers University, Piscataway, NJ 08854, USA; d FutureNeuro, Science Foundation Ireland Research Centre for Chronic and Rare Neurological Diseases, Royal College of Surgeons in Ireland, University of Medicine and Health Sciences, Dublin D02 YN77, Ireland

**Keywords:** Epilepsy, Purinergic signaling, Adenosine triphosphate, Adenosine, P1 receptors, P2 receptors

## Abstract

Despite continuous advances in understanding the underlying pathogenesis of hyperexcitable networks and lowered seizure thresholds, the treatment of epilepsy remains a clinical challenge. Over one third of patients remain resistant to current pharmacological interventions. Moreover, even when effective in suppressing seizures, current medications are merely symptomatic without significantly altering the course of the disease. Much effort is therefore invested in identifying new treatments with novel mechanisms of action, effective in drug-refractory epilepsy patients, and with the potential to modify disease progression. Compelling evidence has demonstrated that the purines, ATP and adenosine, are key mediators of the epileptogenic process. Extracellular ATP concentrations increase dramatically under pathological conditions, where it functions as a ligand at a host of purinergic receptors. ATP, however, also forms a substrate pool for the production of adenosine, via the action of an array of extracellular ATP degrading enzymes. ATP and adenosine have assumed largely opposite roles in coupling neuronal excitability to energy homeostasis in the brain. This review integrates and critically discusses novel findings regarding how ATP and adenosine control seizures and the development of epilepsy. This includes purine receptor P1 and P2-dependent mechanisms, release and reuptake mechanisms, extracellular and intracellular purine metabolism, and emerging receptor-independent effects of purines. Finally, possible purine-based therapeutic strategies for seizure suppression and disease modification are discussed.

## 1 Epilepsy and purinergic signaling

Epilepsy encompasses a heterogeneous group of brain disorders characterized by the manifestation of spontaneous, unprovoked seizures, with an incidence of ~1%, affecting up to 70 million persons worldwide (Thijs et al. 2019). Along with unprovoked seizures, the quality of life of persons with epilepsy is further reduced by a risk of death 2–3 times higher than amongst the general population, and a 4-fold increased risk of additional comorbidities, such as anxiety or depression (LaFrance et al. 2008; Lin et al. 2012; Moshe et al. 2015). Epilepsy can result from genetic abnormalities such as polymorphisms, copy number variations, or *de novo* mutations. Epilepsy can also result from insults to the brain, such as traumatic brain injury (TBI), stroke, prolonged seizures, or infection (Scheffer et al. 2017; Klein et al. 2018).

Despite the heterogeneous nature of epilepsy, research on purinergic signaling and epilepsy has mainly focused on the study of acquired epilepsies, in particular temporal lobe epilepsy (TLE). Among the acquired epilepsies, TLE is the most common form which can result from a precipitating insult to the brain (Klein et al. 2018). TLE pathology includes structural, physiological, biochemical, and epigenetic alterations within the limbic system, including the amygdala and hippocampus (Engel, 1996; Klein et al. 2018). TLE typically involves hippocampal sclerosis, characterized by selective loss of neurons within the hilus and pyramidal neurons of the CA1 and CA3 hippocampal subfields, axonal sprouting and gliosis (Chang and Lowenstein, 2003). Epileptogenesis, triggered by a precipitating insult to the brain, is the process that turns a normal brain into a brain expressing spontaneous epileptic seizures. It is now understood, however, that pathological processes activated during epileptogenesis continue beyond the emergence of epilepsy and lead to an aggravation of the epileptic phenotype over time (Pitkanen and Engel, 2014). Epileptogenesis includes neurodegeneration, changes in structural and synaptic plasticity, increased permeability of the blood-brain barrier (BBB), aberrant neurogenesis, epigenetic changes (*e. g.*, aberrant methylation of DNA, histone modifications) and the chronic activation of inflammatory processes (Henshall and Kobow, 2015; Pitkanen et al. 2015).

While several alternative therapeutic options are available for epilepsy, including surgical resection of the epileptic focus, use of the ketogenic diet, or vagus nerve or deep brain stimulation, for the majority of patients with epilepsy, anti-seizure drugs (ASDs) are the first line treatment (Moshe et al. 2015; Thijs et al. 2019). Current pharmacological interventions in epilepsy, however, remain mostly focused on targeting synaptic transmission or ion channels (Bialer and White, 2010). With over 30 % of patients not responding to current medication with a high burden of adverse effects (*e.g.*, fatigue, irritability, dizziness), currently available medications are merely symptomatic, without noticeable impacts on disease progression. There is, therefore, a big need to better understand the processes that lead to epilepsy and its progression and to develop new therapies that go beyond seizure control (Loscher, 2020).

Over recent years, the role played by the purinergic signaling system in mediating the excitability of neuronal networks has become increasingly clear. It is now well established that neuronal excitability in the brain is determined not only by the glutamate/γ-aminobutyric acid (GABA) balance, but also by the ATP/adenosine balance. That adenosine functions as an endogenous anticonvulsant and seizure terminator has been known for decades (Dunwiddie and Hoffer, 1980; Dragunow and Goddard, 1984), while the excitatory role played by ATP during seizures has emerged more recently (Engel et al. 2012). The contribution of purines to the development and progression of epilepsy, however, is not restricted to their function at the synapse, but also in mediating other biological processes, such as inflammation (Beamer et al. 2016) or newly identified epigenetic functions of adenosine (Williams-Karnesky et al. 2013; Boison and Rho, 2019). New evidence has recently emerged, clarifying the cellular sources, initiating stimuli, and mechanisms of release of purines into the extracellular space following an insult (Beamer et al. 2019). Further, it is important to consider the role played by ectoenzymes in regulating the extracellular concentrations of both ATP and adenosine, and the interactions between extra- and intracellular adenosine metabolism, as well as transport (Plesner, 1995; Boison and Yegutkin, 2019). The two signaling systems of ATP and adenosine are intrinsically linked and a full understanding requires the purinergic signaling system to be considered as a whole. In the following section, we will provide a detailed discussion of recent advances in our understanding of purinergic signaling during seizures and epilepsy, including the release and degradation of purines and the contribution of the different purinergic receptor subtypes to disease pathology. We will also discuss how purinergic therapeutic strategies could be used to suppress seizures and prevent the development of epilepsy.

## 2. The role of purines in seizures and epilepsy

Over the past decades our understanding of the role played by purinergic signaling during seizures and epilepsy has increased substantially. While most efforts have been put on investigating a contribution of purines to status epilepticus and TLE using rodent models of chemically-induced status epilepticus [e.g., pilocarpine, kainic acid (KA)], other models mimicking a variety of different seizure types and epilepsies are also used including genetic models (e.g., Wistar Albino Glaxo/Rijswijk (WAG/Rij) rats) and electrostimulation models (Table 1). For a comprehensive and exhaustive description of seizure and epilepsy models please refer to other excellent reviews which were written on this topic [e.g., (Loscher, 2017; Campos et al. 2018; Loscher, 2020)].

### 2.1. Release of purines during acute CNS insults and seizures

**Fig. 1.**
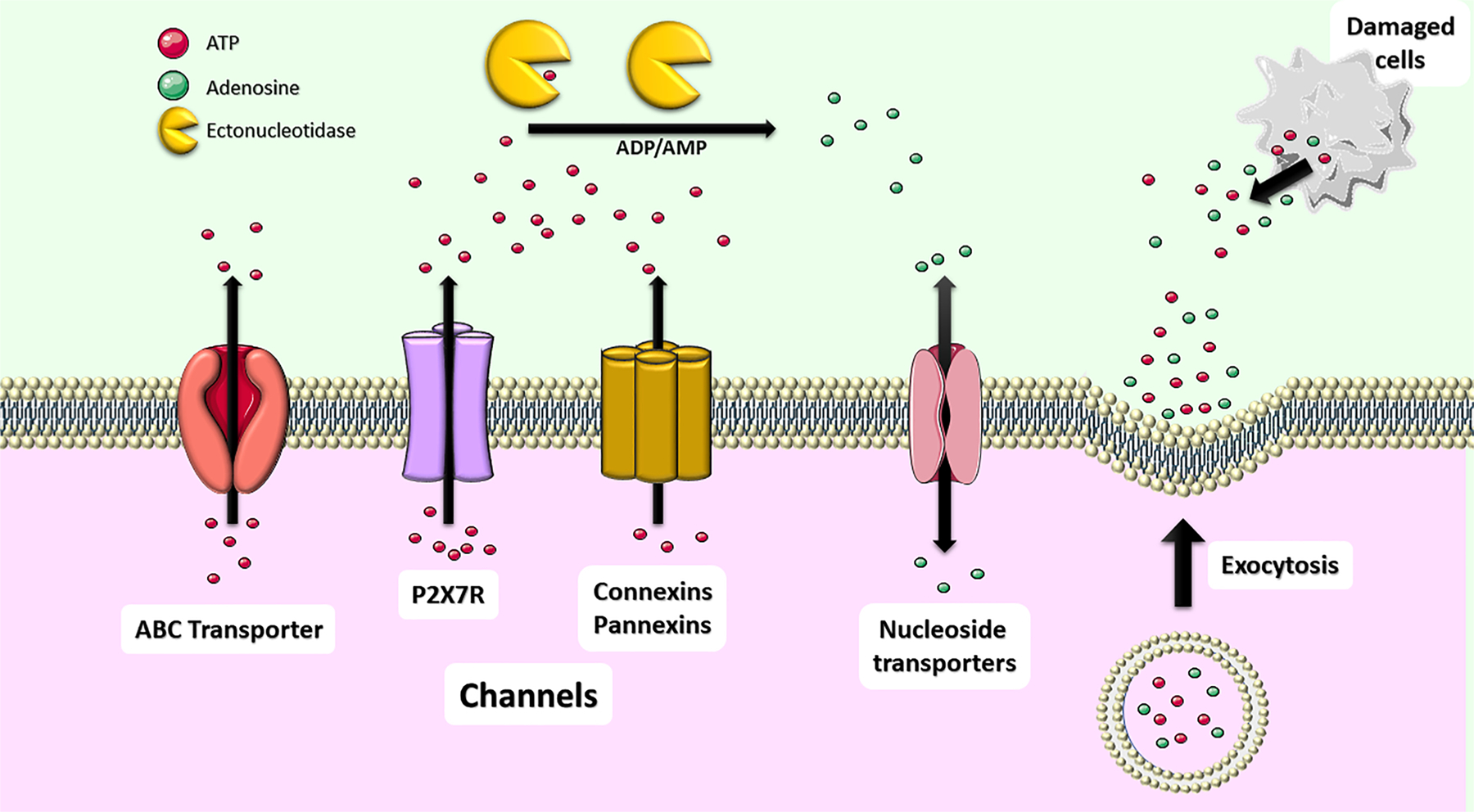
Sources of extracellular purines. (A) Purines (ATP and adenosine) can be released into the extracellular space from both neurons and glia via exocytosis or non-exocytotic mechanisms including transporters (*e.g.*, ATP-binding cassette (ABC) transporters for ATP and Nucleoside Transporters such as equilibrative nucleoside transporter (ENT) and concentrative nucleoside transporter (CNT) for adenosine) and membrane channels such as the P2X7R, and Pannexin-1 and Connexin-43 hemichannels. Purines are also released from dying cells escaping across a compromised lipid bilayer acting thereby as a danger signal. Once released, ATP is metabolized into different breakdown products including ADP, AMP and adenosine via the action of different ectonucleotidases as discussed in Chapter 5.

Evidence for purine release into the extracellular space (Fig. 1) during and following acute central nervous system (CNS) insults comes from *in vitro* preparations, *in vivo* models, and clinical measurements (Dale and Frenguelli, 2009). Overall, evidence for peri-insult spikes in extracellular adenosine is stronger than for ATP. Independent of the insult or assay, where both purines are measured simultaneously, increases in extracellular adenosine are invariably of a greater magnitude than that of ATP. Further, peri-insult spikes in extracellular adenosine have been demonstrated in a wider range of contexts than for ATP. The contribution of the extracellular pool of ATP to adenosine, via extracellular metabolism has long been debated, however, the consensus is that insult-induced spikes in extracellular adenosine are largely the result of direct release of adenosine from cells (Wall and Dale, 2008).

Enzyme-based sensors have been used to demonstrate release of both adenosine (Dale et al. 2000) and ATP (Frenguelli et al. 2007) in *in vitro* models of cerebral ischemia. The same sensors have been used to demonstrate adenosine release following *in vitro* seizure models (Diez et al. 2017). Similar studies investigating ATP release in the same context, however, have been mixed (Beamer et al. 2019) and appear to be dependent on the method of seizure induction employed. While seizure-like events induced by high K^+^ initiate ATP release (Heinrich et al. 2012), other methods, such as stimulation of the Schaffer collateral (Lopatar et al. 2011) found no increase in extracellular ATP. Diez *et al.* (Diez et al. 2017) found that ictal activity induced by low extracellular Ca^2+^ led to an increase in adenosine release through nucleoside transporters, but no change in extracellular ATP concentrations. The strongest *in vitro* evidence for ATP release during seizures comes from a study performed using post-operative brains from drug-resistant epilepsy patients (Dossi et al. 2018). In this study, extracellular ATP increased 80 % during high K^+^-induced ictal discharges. Similarly treated non-ictogenic surrounding tissue showed no ictal activity in response to high K^+^ and no ATP release. Further, ATP release, but not ictal activity, was blocked by pharmacological blockade of the Pannexin-1 hemichannel. It is not currently clear why high K^+^ *in vitro* seizure models induce ATP release, but other preparations do not.

Sims & Dale (Sims and Dale, 2014) report that adenosine is released from highly active cells as a consequence of increased activity of the Na^+^-K^+^-ATPase enzyme, necessary for the maintenance of ion homeostasis across the cell membrane. As the enzyme depletes ATP in order to drive ions against their concentration gradient, the intracellular concentration of adenosine increases, driving its transport across the cell membrane into the extracellular space through nucleoside transporters (Diez et al. 2017).

Following an insult, purines can enter the extracellular milieu via disruption of the integrity of cellular membranes, with ATP in particular driven by a very high intracellular-extracellular concentration gradient (Seminario-Vidal et al. 2009). Non-lytic routes of purine release, however, are many and varied. Wall & Dale (Wall and Dale, 2013) argue that under excitotoxic conditions, adenosine is released from neurons, while ATP is released from astrocytes, with the subsequent extracellular ATP pool then contributing to adenosine production via ectoenzymes. ATP release has been described via secretory granules from glia cells (Imura et al. 2013; Lalo et al. 2014), neuronal synaptic vesicles (Jo and Schlichter, 1999), diffusion through pannexin-1 hemichannels in neurons (Xia et al. 2012) and astrocytes (Bennett et al. 2012) and through connexin-43 hemichannels in astrocytes (Orellana et al. 2011). ATP is also released through the ATP-activated purinergic P2X7R (Suadicani et al. 2006). The first evidence for purine release from *in vivo* models of acute brain insult was produced by Berne et al. (Berne et al. 1974) demonstrating that interstitial adenosine increases 3-fold in the brains of dogs and rats within a minute of experimental ischemia. More recently, Ganesana & Venton (Ganesana and Venton, 2018) used fast-scan cyclic voltammetry to demonstrate an increased frequency of adenosine transients following experimental cerebral ischemia (bilateral common carotid artery occlusion) in the rat, with a 1.5-fold increase in cumulative concentration. Dona et al. (Dona et al. 2016), meanwhile, demonstrate that, in a rat model of pilocarpine-induced status epilepticus, adenosine is increased approximately 3-fold, alongside adenosine monophosphate (AMP) (2.5 fold) and adenosine diphosphate (ADP) (3.5-fold), with no measurable increase in ATP.

The direct measurement of ATP release *in vivo* is more challenging than adenosine, for a number of reasons. Firstly, it is present at extremely low baseline absolute extracellular concentrations, close to the threshold of detection (Dona et al. 2016). Secondly, the concentration gradient between the intracellular and extracellular compartments is around 1,000,000:1 (Schwiebert and Zsembery, 2003), meaning that cell damage resulting from invasive recording methods is likely to introduce experimental artifacts with a magnitude far in excess of the signal. Thirdly, ATP has a half-life in the extracellular milieu on the order of seconds (Picher et al. 2004), where it is swiftly degraded by a coordinated array of ectoenzymes (Yegutkin et al. 2006). Despite these challenges, convincing *in vivo* evidence has been presented for increases in interstitial ATP concentrations following a model of cerebral ischemia in the rat, with interstitial ATP increasing 1.3-fold (Melani et al. 2005). Evidence for ATP release during seizures, however, is more complex (Beamer et al. 2019).

Early *in vivo* evidence for seizure-induced ATP release comes from studies demonstrating a 30-fold spike in ATP measured in extracellular fluid collected in a cup from the cortex following direct electrical stimulation (Wu and Phillis, 1978). Haman & Attwell (Hamann and Attwell, 1996) suggest, however, that these findings may be an artefact of electroporation of cells. More recently, two studies have been performed using the lithium-pilocarpine model in rats (Dona et al. 2016; Lietsche et al. 2016). These studies found no change in extracellular concentrations of ATP in the hippocampus during or following status epilepticus. Lietsche et al. (Lietsche et al. 2016), however, found an 8-fold increase in extracellular ATP vs. controls, when an ectoATPase inhibitor was introduced with the microdialysis probe. This suggests that seizure activity increases both the cellular release of ATP into the extracellular space and the rate of its enzymatic degradation. Dona et al. (Dona et al. 2016), meanwhile, demonstrate, that while no ATP increase could be observed during acute, induced seizures, spontaneous seizures during the chronic phase of epilepsy were accompanied by a 4-fold increase in interstitial ATP concentration.

Clinical evidence for insult-driven purine release is restricted to adenosine and adenosine metabolites. Adenosine is elevated in the cerebrospinal fluid (CSF) in children following TBI (Robertson et al., 2001), correlating strongly with severity on the Glasgow Coma Scale. Bell et al. (Bell et al., 2001) report that interstitial concentrations of adenosine increase 3.1-fold following TBI in adults, along with concomitant increases in xanthine, hypoxanthine and cyclic AMP (cAMP). Dale et al. (Dale et al. 2019) report a 1.6-fold increase in adenosine concentration in the venous blood of stroke patients, within 4.5 h of symptom onset. During & Spencer (During and Spencer, 1992), meanwhile, recorded directly from the brain of an epilepsy patient during a seizure, using a deep electrode and microdialysis probe. Extracellular concentrations of adenosine increased 6 – 31-fold during a seizure and persisted into the post-ictal period, a concentration of adenosine the authors demonstrated to be sufficient for the suppression of ictal-like activity in *in vitro* models.

### 2.2. Purinergic receptors

#### 2.2.1. P1 (Adenosine) receptors

The family of G protein-coupled purinergic P1 receptors, also known as adenosine receptors (ARs), is comprised of four subtypes: A_1_,R A_2A_R, A_2B_R, A_3_R, which have all been cloned and extensively characterized (Dunwiddie et al. 1997; B.B. Fredholm et al., 1999; Benarroch, 2008). ARs are widely distributed throughout the nervous, respiratory, cardiovascular, immune, gastrointestinal, and urogenital systems, and are also found in skin, bones, and eyes (Peleli et al. 2017). Each AR varies, not only in distribution across tissue and cell type, but also in terms of downstream effectors and physiological effects they mediate. A_1_Rs and A_3_Rs are coupled to inhibitory G_i/o_ proteins, whereas A_2A_Rs and A_2B_Rs are coupled to stimulatory G_s_ proteins (Borea et al. 2018). All ARs are activated by their endogenous ligand, adenosine. The affinity of each receptor, however, may vary depending on the assay systems used (Fredholm, 2014). Based on current consensus, adenosine has a high affinity for A_1_Rs, A_2A_Rs, and A_3_Rs as compared to the A_2B_R subtype, with affinities of 1–10 nM, 30 nM, 100 nM and 1000 nM, respectively (Fredholm et al., 2011; Borea et al. 2018).

#### 2.2.2. P2 (ATP) receptors

P2 receptors, activated by adenine and uridine nucleotides are divided into two groups based on their sequence homology, pharmacology and mechanism of action; the ionotropic P2X receptors (P2XR) and metabotropic P2Y receptors (P2YR) which are expressed and functional on both neurons and glial cells (Burnstock, 2016a,b). The fast-acting P2XRs, all activated by their main endogenous agonist, ATP, are a family of cation-selective channels, permeable to small cations including Na^+^, K^+^ and Ca^2+^. To date, seven mammalian subunits have been cloned (P2X1-7), ranging in length from 379 (P2X6) to 595 (P2X7) amino acids (Khakh and North, 2006). These subunits form either functional homo- or heterotrimers, depolarizing the cell membrane upon activation. P2XRs share a common topology with two transmembrane domains, a large extracellular loop and an intracellular amino and carboxyl terminus (Khakh and North, 2006). P2XRs are expressed throughout the brain and are present and functional on all cell types. Some controversy remains, however, regarding the cell type-specific expression and function of some receptors, in particular regarding the P2X7R subtype (Illes et al. 2017; Miras-Portugal et al. 2017). In the brain, the contribution of P2XRs to synaptic plasticity and, particularly, fast synaptic transmission has been well-established (Pankratov et al. 2009). The role of P2XRs is not limited, however, to the regulation of synaptic transmission. P2XRs have also been implicated in numerous other cellular processes such as proliferation, differentiation, maturation and survival, cell adhesion, migration and inflammation (Burnstock, 2016a,b).

The slower-acting metabotropic P2YR family consists of eight G-protein coupled receptors including P2Y_1_, P2Y_2_, P2Y_4_, P2Y_6_, P2Y_11_, P2Y_12_, P2Y_13_ and P2Y_14_. P2YRs feature the typical characteristics of G-protein-coupled receptors, including an extracellular amino terminus, intracellular carboxyl terminus and seven transmembrane-spanning motifs. While P2Y_1_, P2Y_2_, P2Y_4_, P2Y_6_ and P2Y_11_Rs are coupled to Gq/G_11_ proteins, stimulating phospholipase C leading to the release of calcium from intracellular stores and the activation of protein kinase C (PKC), P2Y_12_, P2Y_13_ and P2Y_14_Rs are coupled to G_i/_G_0_ proteins decreasing cAMP production via inhibition of adenylate cyclase. The P2Y_11_R is an exception as this receptor also couples to Gs, increasing the production of cAMP via stimulation of adenylate cyclase. Endogenous agonists of the P2YR family include the adenine nucleotides ATP (P2Y_1_R, P2Y_2_R and P2Y_11_R) and ADP (P2Y_1_R, P2Y_12_R and P2Y_13_R) and the uridine nucleotides uridine triphosphate (UTP) (P2Y_2_R and P2Y_4_R), uridine diphosphate (UDP) (P2Y_6_R and P2Y_14_R) and UDP-glucose (P2Y_14_R) (von Kugelgen and Hoffmann, 2016; von Kugelgen, 2019). As for P2XRs, P2YRs are expressed and functional on all brain cells and are implicated in numerous different cellular functions and pathological processes pertinent to epileptogenesis and epilepsy. These range from synaptic reorganization, changes in neurotransmitter release, cellular survival and neuroinflammation (Jacobson and Boeynaems, 2010; Guzman and Gerevich, 2016).

### 2.3. The role of adenosine P1 receptors in seizure modulation and epilepsy

#### 2.3.1 A_1_ receptors

A_1_Rs interact with G_i_ and G_o_ proteins, contributing to the suppression of adenyl cyclase and a consequent reduction in the production of cAMP, reduction in the activation of protein kinase A (PKA), and inhibition of GABA uptake into astrocytes (Fredholm et al., 2011; Cristovao-Ferreira et al. 2013). The A_1_R subtype is widely expressed in the CNS, mainly in pre- and post-synaptic excitatory synapses of the cortex, hippocampus, cerebellum, spinal cord, in glial cells, and autonomic nerve terminals (Chen et al. 2013). Activation of A_1_Rs mediates a variety of different functions including reduction of neuronal hyperexcitability, neuroprotection, seizure control, pain reduction, and control of sleep/-wakefulness (Stenberg et al. 2003; Ciruela et al. 2006; Gessi et al. 2011; Cunha, 2016; Sawynok, 2016). The role of A_1_Rs on physiological neurotransmission is through the blockade of N-type calcium channels and the induction of neuronal hyperpolarization through activation of G-protein coupled inwardly rectifying potassium (GIRK) channels (Wu and Saggau, 1994; Gundlfinger et al. 2007). This leads to a combined action of presynaptic inhibition of neurotransmitter release (glutamate) and decreased postsynaptic glutamate receptor (N-methyl-D-aspartate receptor (NMDA)) activation, thereby suppressing neuronal hyperexcitability and maintaining an A_1_R-dependent inhibitory tonus in the brain (Yoon and Rothman, 1991; Von Lubitz et al. 1994). The anti-convulsant effects of adenosine are largely mediated by A_1_Rs, because of this receptor’s high affinity for adenosine and the dominance of its expression in the seizure-prone limbic system (Hargus et al. 2012). In TLE patients, a 6 to 31-fold increase in adenosine levels and an increase in A_1_R expression was shown following a seizure when compared to baseline (During and Spencer, 1992). The increase in adenosine and consequent A_1_R activation is believed to be a protective feedback mechanism to limit seizure duration and the intensity or spread of focal seizures (Dulla et al. 2009; Ilie et al. 2012; Lovatt et al. 2012; Van Gompel et al. 2014). Furthermore, activation of A_1_Rs through selective receptor agonists effectively suppresses seizure activity, even in pharmaco-resistant epilepsy (Gouder et al. 2003; Vianna et al. 2005; Mares, 2010; Li and Zhang, 2011; Tosh et al. 2012; Li et al. 2013; Muzzi et al. 2013). Importantly, a local increase in adenosine resulting in enhanced A_1_R activation in the hippocampus is sufficient to exert potent anticonvulsant effects, while avoiding systemic side-effects associated with global A_1_R activation (Huber et al. 2001; Guttinger et al. 2005). The importance of A_1_Rs has been demonstrated in A_1_R-knock-out (KO) mice, which not only display spontaneous electrographic seizures (Masino et al. 2011), but also a generalization and spread of focal seizure activity after unilateral intrahippocampal KA-induced status-epilepticus (Fedele et al. 2006). A_1_R-KO mice also exhibited excessive neuronal cell loss when subjected to seizures (Fedele et al. 2006) and TBI (Kochanek et al. 2006). Findings from those studies highlight *(i)* the capability of endogenous adenosine to limit seizure activity to an epileptogenic focus and to prevent propagation of seizure activity to other brain regions and *(ii)* the neuroprotective effects associated with A_1_R activation (Fedele et al. 2006; Kochanek et al. 2006). In line with these findings, A_1_R antagonists increase seizure activity and decrease the potential benefits of standard ASDs (Chwalczuk et al. 2008; Fukuda et al. 2010). In spite of the anti-convulsant and neuroprotective properties of adenosine and A_1_R activation, it is important to note that the seizure-induced increase in endogenous adenosine might also be implicated in sudden unexpected death in epilepsy (SUDEP), because of the depressant effect of adenosine on brainstem respiratory centers (Vandam et al. 2008; Faingold et al. 2016; Reklow et al. 2019).

#### 2.3.2. A_2_ and A_3_ receptors

In epilepsy, overexpression of A_2A_Rs in the cerebral cortex was found in both amygdala-kindled rats and following systemic KA-induced status epilepticus in mice, with upregulation largely restricted, in both cases, to excitatory glutamatergic terminals (Rebola et al. 2005). In addition, there is a threefold increase in A_2A_Rs in hippocampal astrocytes of patients with mesial temporal lobe epilepsy (MTLE), when compared to controls (Barros-Barbosa et al., 2016a). Interestingly, no A_2A_R upregulation was seen in GABAergic synapses, which would imply that A_2A_Rs function largely through their mediation of excitatory neurotransmission (Canas et al. 2018).

A_2A_Rs are coupled to G_s_ proteins and are associated with the activation of adenylate cyclase (Kull et al. 2000). Contrary to A_1_Rs, A_2A_Rs are known for their excitatory effects through the enhancement of NMDA receptor function and increase of glutamate release at glutamatergic axon terminals (Lopes et al. 2002; Marchi et al. 2002; Rebola et al. 2008; Rebola et al. 2011). Although some studies have suggested a neuroprotective and anti-convulsive role of A_2A_Rs (Adami et al. 1995; Vianna et al. 2005), the majority of available data demonstrates a proconvulsive and neurodegenerative role for A_2A_Rs (Stockwell et al. 2017). In a recent study, A_2A_Rs were shown to play a key role in neurodegeneration via the modulation of synaptic excitability following KA-induced seizures in rats and mice (Canas et al. 2018). The sequence of events observed are, an initial process of glutamate-induced excitotoxicity at 6 h, followed by synaptotoxicity at 12 h, and finally overt neurodegeneration at 24 h, suggesting a window of opportunity for intervention to achieve seizure suppression by using A_2A_R antagonists (Canas et al. 2018). In support of the pro-convulsant action of A_2A_Rs, mice with a genetic inactivation of A_2A_Rs were shown to have a reduced seizure susceptibility (El Yacoubi et al. 2001, 2008; El Yacoubi et al. 2009). The robust increase in A_2A_R- and decrease in A_1_R-density over time provides a rationale for a combined approach using A_2A_R antagonism and A_1_R agonism for seizure control (Rebola et al. 2005). The roles of A_2B_ and A_3_Rs in epilepsy are not clearly characterized. Activation of A_3_Rs can counteract the inhibitory effects of A_1_Rs through heterologous desensitization of A_1_Rs (Dunwiddie et al. 1997) and A_3_R activation by endogenous adenosine increased epileptic activity in hippocampal slice cultures (Etherington and Frenguelli, 2004), possibly through interaction with A_1_Rs. On the contrary, A_3_R antagonism decreased seizure intensity and duration *in vitro* (Etherington and Frenguelli, 2004). Both A_2B_ and A_3_R antagonists reduced GABA currents in membrane preparations derived from human epileptic hippocampi after transplantation into frog oocytes (Roseti et al. 2008).

Taken together, while the anticonvulsant effects of adenosine are well-established, we have now an increasing understanding of how adenosine receptors function during seizures and epilepsy, critical to develop adenosine-based therapeutic strategies for seizure control and epilepsy (Table 2).

### 2.4. The role of ATP P2 receptors in seizure modulation and epilepsy

Although the study of adenine and uracil nucleotide-activated P2 receptors in epilepsy is relatively new when compared to adenosine-activated P1 receptors, there has been substantial progress made over the past decade deciphering the role P2 receptors play, not only during seizure generation, but also during the development and maintenance of epilepsy (Engel et al. 2016; Rassendren and Audinat, 2016; Cieslak et al. 2017). Early evidence linking extracellular ATP to increased hyperexcitability stems from studies in a seizure-prone strain of mice, which presented increased extracellular ATP concentrations (Wieraszko and Seyfried, 1989) and from data showing motor seizures caused by microinjections of ATP into the prepiriform cortex (Knutsen and Murray, 1997). More recent evidence demonstrating a contribution of elevated extracellular ATP to brain hyperexcitability was provided by studies showing that direct injection of ATP, ADP or the ATP analog 2’, 3’-O-(4-benzoyl-benzoyl)ATP (B_Z_ATP) leads to high spiking on the electroencephalogram (EEG) or exacerbates seizure activity during intra-amygdala induced-status epilepticus (Engel et al. 2012; Sebastian-Serrano et al., 2016; Alves et al. 2017). In contrast to adenine nucleotides, however, treatment with the uridine nucleotide, UTP, reduced seizure severity during intra-amygdala induced status epilepticus in mice (Alves et al. 2017) and neuronal firing in Long Evans and Wistar Albino Glaxo/Rijswijk (WAG/Rij) rats, both strains modelling absence seizures (Kovacs et al. 2013).

#### 2.4.1. Changes in P2 receptor expression

##### 2.4.1.1. Acute seizures.

Among P2X receptors, changes in expression associated with seizures or a period of status epilepticus have been identified in P2X1R, P2X2R, P2X4R and P2X7R. These findings come from chemoconvulsant-induced status epilepticus models in mice. The P2X1R has been reported to be increased in the hippocampus 24 h following intraperitoneal KA in mice at the transcriptional level (Avignone et al. 2008). No changes in hippocampal P2X1R protein levels have, however, been found using the intra-amygdala KA mouse model (Engel et al. 2012). The P2X2R has been shown to be downregulated 24 h following status epilepticus in the intra-amygdala KA mouse model (Engel et al., 2012), while P2X4R expression was found to be increased in the hippocampus of mice 24 h following systemic administration of KA (Avignone et al. 2008; Ulmann et al. 2013), but not intra-amygdala KA- or pilocarpine-induced status epilepticus (Dona et al. 2009; Engel et al. 2012).

The P2XR which has been studied in most depth in association with seizures is the P2X7R. Under normal physiological conditions, P2X7R expression is mainly restricted to endothelial cells, microglia, oligodendrocytes and neural progenitor cells (Monif et al. 2009; Rozmer et al. 2017; Kaczmarek-Hajek et al. 2018). It is also suggested, however, that the P2X7R is expressed in neuronal presynaptic termini (Armstrong et al. 2002; Sperlagh et al. 2006). Following status epilepticus, P2X7R is consistently upregulated at both the transcriptional and expressional level. *P2rx7* mRNA is elevated in the hippocampus following either intraperitoneal or intra-amygdala KA-induced status epilepticus in mice (Avignone et al. 2008; Engel et al. 2017). At the protein level, P2X7R is increased post-status epilepticus in the hippocampus and cortex of rats and mice subjected to pilocarpine- and KA-induced status epilepticus. While some studies using systemic KA-treated mice and pilocarpine-treated rats have shown that P2X7R is mainly up-regulated on microglia in both cortex and hippocampus shortly following status epilepticus (Rappold et al. 2006; Kim et al. 2009), thereby possibly contributing to microglia activation (Avignone et al. 2008), other studies have also suggested a neuronal upregulation post-intra-amygdala KA-induced status epilepticus (Dona et al. 2009; Engel et al. 2012). P2X7R expression was also found to be increased on neuronal progenitor cells following status epilepticus induced via systemic KA in mice (Rozmer et al. 2017), potentially impacting on status epilepticus-induced aberrant neurogenesis. Neuronal expression of the P2X7R has, however, recently been questioned using a P2X7R reporter mouse, where P2X7R is fused to the enhanced green fluorescent protein (EGFP) (Kaczmarek-Hajek et al. 2018). In this model P2X7-EGFP co-localized to microglia and oligodendrocytes post-intra-amygdala KA-induced status epilepticus in mice, however, no co-localization was observed for P2X7-EGFP with neurons or astrocytes (Morgan et al. 2020). Thus, while there is broad consent for P2X7R upregulation on microglia following status epilepticus, the question of whether the P2X7R increases on neurons is still a matter of debate. Progress has also been made in deciphering what drives P2X7R expression in the brain during seizures. Following intra-amygdala KA induced status epilepticus in mice, P2X7R transcription seems to be under the control of the specificity protein 1 (Sp1) (Engel et al. 2017) which is a member of the injury-activated transcription factor (IATF) family and highly expressed in the brain (Kiryu-Seo et al. 2008). Using the same intra-amygdala KA mouse model, P2X7R expression during status epilepticus also appears, however, to be controlled at the post-transcriptional level, involving the targeting of *P2rx7* mRNA by microRNA-22, which is also under the control of Sp1 (Engel et al. 2017). Increased microRNA-22 expression under the control of Sp1 is highly sensitive to intracellular Ca^2+^ concentrations. Elevated Ca^2+^ concentrations associated with status epilepticus block Sp1 binding to the microRNA-22 promotor, disinhibiting the suppression of *P2rx7* mRNA translation into protein (Engel et al. 2017). This suggests that, while ubiquitously expressed in all cell types, Sp1 is capable of modulating its activity to provide a stimulation and/or injury-specific transcriptional response, thereby possibly contributing to the cell type-specific expression profile of down-stream targets such as the P2X7R. Of note, Sp1 also contributes to the regulation of the *Adk* gene, providing a possible mechanisms to link P2X7R signaling to adenosine metabolism (Kiese et al. 2016).

One of the first studies analyzing changes in the expression of the P2YR family following status epilepticus was carried out using the intraperitoneal KA mouse model. Here the authors observed increased transcription of *P2ry*_*6*_, *P2ry*_*12*_ and *P2ry*_*13*_ in the hippocampus (Avignone et al. 2008). Rozmer et al. (Rozmer et al. 2017) showed the P2Y_1_R, similar to the P2X7R, to be upregulated on neuronal progenitor cells following pilocarpine-induced status epilepticus in mice. Using the intra-amygdala KA and intraperitoneal pilocarpine mouse model of status epilepticus, Alves et al. carried out the first comprehensive study analyzing the expression of the entire P2YR family post-status epilepticus (Alves et al. 2017). Analyzing the hippocampus at different time-points following status epilepticus, the authors found that while mRNA levels of the uridine-sensitive P2Y_2_R, P2Y_4_R and P2Y_6_R were increased, transcription of the adenine-sensitive P2Y_1_R, P2Y_12_R and P2Y_13_R was downregulated. On the other hand, at the protein level, P2YRs coupled to Gq showed an increase in their expression (P2Y_1_R, P2Y_2_R, P2Y_4_R and P2Y_6_R), while P2YRs coupled to Gi were either down-regulated (P2Y_12_R) or unchanged (Alves et al. 2017). These surprising results suggest that changes in P2YR transcription and expression in the hippocampus post-status epilepticus are dependent on P2YR agonists (*e.g.,* uridine-sensitive receptors show increased transcription) and downstream signaling (*e.g.,* P2Y receptors coupled to Gq show increased expression). In contrast to hippocampal P2YR expression, P2YR expression in the cortex was mainly upregulated following intra-amygdala KA in mice, with P2Y_1_R and P2Y_4_R showing the strongest increase (Alves et al., 2019b).

##### 2.4.1.2. Epilepsy.

Unlike seizures or status epilepticus, clinical evidence is available for changes in P2 receptor expression during epilepsy, with tissue available from surgical resection from patients with drug resistant TLE and focal cortical dysplasia. Whereas most widely reported changes in receptor expression have been found for the P2X7R (Beamer et al. 2017), other P2XRs analyzed during epilepsy include the P2X2R, P2X3R and P2X4R. While both P2X2R and P2X4R expression was found to be decreased in the hippocampus of seizure-sensitive gerbils (Kang et al. 2003), P2X4R expression was also found to be decreased in the hippocampus of pilocarpine-treated epileptic rats (Dona et al. 2009). In contrast, P2X3R, has been found increased in the cortex of patients with TLE and in the hippocampus and cortex of epileptic pilocarpine-treated rats being mainly localized to the cell bodies and dendrites of neurons (Zhou et al. 2016). P2X7R is increased in the hippocampus and cortex during epilepsy in both experimental rodent models of TLE and patients with TLE (Dona et al. 2009; Jimenez-Pacheco et al. 2013; Barros-Barbosa et al., 2016b; Jimenez-Pacheco et al. 2016). Early studies analyzing changes in P2X7R expression during epilepsy using a pilocarpine rat model demonstrated an increase in the hippocampus, with strong immunoreactivity associated with microglia, mossy fibers and glutamatergic nerve terminals (Vianna et al. 2002; Dona et al. 2009). Similarly to status epilepticus, more recent studies seem to confirm a mainly microglial and neuronal P2X7R induction during epilepsy in the hippocampus and cortex using the intra-amygdala KA mouse model (Jimenez-Pacheco et al. 2013; Jimenez-Pacheco et al. 2016). These data were, however, challenged by a study using P2X7-EGFP reporter mice subjected to intra-amygdala KA showing P2X7R expression increases to be restricted to microglia and oligodendrocytes (Morgan et al. 2020). Increases in P2X7R expression were absent on astrocytes during experimental epilepsy (Jimenez-Pacheco et al. 2016; Morgan et al. 2020).

Much less is known regarding the expression of P2YRs during epilepsy. Using the intra-amygdala KA mouse model, Alves et al. (Alves et al. 2017) showed that, in contrast to status epilepticus, hippocampal P2YR transcription and expression is either increased (mRNA: *P2ry*_*1*_, *P2ry*_*2*_ and *P2ry*_*6*_; protein: P2Y_1_R, P2Y_2_R and P2Y_12_R) or remains unchanged during epilepsy, suggesting hippocampal P2YR upregulation to be the predominant response during experimental epilepsy. P2YR expression is also increased in resected tissue from patients with TLE with the only exception being the P2Y_13_R, which is downregulated (Alves et al. 2017). Further proof of P2YR upregulation in the hippocampus during epilepsy is provided in an investigation carried out by Sukigara et al. where the authors found increased levels of P2Y_1_R, P2Y_2_R and P2Y_4_R in hippocampal tissue from patients with intractable epilepsy associated with focal cortical dysplasia, with the main increase in astrocytes (Sukigara et al. 2014).

#### 2.4.2. Targeting P2 receptors during seizures and epilepsy

##### 2.4.2.1. Targeting P2XRs during acute seizures.

As mentioned in the previous paragraph, among the P2XRs, the P2X7R subtype has attracted most attention as a potential drug target in epilepsy (Beamer et al. 2017). Among the P2XR family, P2X7R has some unique structural and functional characteristics, making this receptor a particularly attractive therapeutic target. It has a relatively low affinity for ATP (EC_50_ ≥ 100 μM, activation threshold: 0.3–0.5 mM), suggesting P2X7R activation occurs mainly under pathological conditions of high ATP release. It also has slow desensitization dynamics, the ability to permeabilize the cell membrane to molecules up to 900 Daltons in size and is a key driver of inflammation via activation of the NLRP3 inflammasome (Beamer et al. 2016; Adinolfi et al. 2018). For a detailed review on P2X7R function in the brain see (Jimenez-Mateos et al. 2019; Kopp et al. 2019). The first data suggesting anticonvulsive properties of P2X7R antagonists during status epilepticus was provided using the intra-amygdala KA mouse model. Here, mice treated before and shortly following intra-amygdala KA with the P2X7R antagonists A-438079 and Brilliant Blue G (BBG) (pre-and post-treatment regime) experienced less severe seizures (EEG and behavioral) and a reduction in neurodegeneration (Engel et al. 2012; Jimenez-Pacheco et al. 2013). In the same study, seizure severity was also reduced in P2X7R KO mice (Solle et al. 2001) and by P2X7R antibodies delivered into the lateral ventricle (Engel et al., 2012). Critically, P2X7R antagonists, when given in combination with lorazepam at a later time-point during status epilepticus, when sensitivity to lorazepam was reduced, efficiently stopped seizures, suggesting the potential of P2X7R antagonists as adjunctive treatment for pharmaco-resistant status epilepticus (Engel et al. 2012). Treatment using three different P2X7R antagonists (BBG, A-438079, A-740003) reduced seizures also during coriaria lactone-induced status epilepticus in rats (Huang et al. 2017).

Other studies, however, only observed limited or no effect via P2X7R antagonism. Nieczym et al. (Nieoczym et al. 2017) only found a weak anticonvulsant potential via the P2X7R antagonist BBG in the intravenous PTZ seizure threshold, maximal electroshock seizure threshold and 6 Hz psychomotor seizure threshold tests. In line with this, Fischer et al. observed no anticonvulsant effects provided by P2X7R antagonism using four different antagonists (JNJ-47965567, AFC-5128, BBG, transhinone IIA sulfonate) in the maximal electroshock seizure threshold test and the PTZ seizure threshold test in mice, although when given in combination with carbamazepine, P2X7R antagonists JNJ-47965567 and AFC-5128 increased the threshold in the maximal electroshock seizure test (Fischer et al. 2016). In a recent study, Dogan et al. (Dogan et al. 2020) using WAG/Rij rats, a model of absence seizures, observed no anticonvulsive effects when treating rats with the P2X7R antagonist A-438079.

In contrast to a pro-convulsive function of the P2X7R during seizures, Kim et al. found that P2X7R deficiency exacerbated seizure severity in the intraperitoneal pilocarpine mouse model (Kim and Kang, 2011). Moreover, P2X7R antagonism via oxidized ATP (OxATP) and BBG protected against astroglial cell death (Kim et al. 2009) and reduced infiltration of neutrophils into the frontoparietal cortex in the pilocarpine rat model (Kim et al. 2010). The same group further showed that treatment with the P2X7R antagonists A-438079, A-740003 and OxATP increased hippocampal cell death in the pilocarpine rat model of status epilepticus (Kim et al. 2011). P2X7R deficiency did, however, not alter seizure severity in the systemic KA mouse model or the picrotoxin mouse model (Kim and Kang, 2011).

Taken together, while the P2X7R seems to play a role during prolonged, damaging seizures (*i.e.*, status epilepticus) with P2X7R antagonists altering seizure severity and seizure-induced pathology, effects of P2X7R antagonism seem to be minor or absent during acute non-damaging seizures (*e.g.*, genetic model and electrical induced seizures). This suggests that P2X7R-based treatment is most likely more effective in reducing seizures once pathological processes have been triggered (*e.g.*, increased inflammation during drug-refractory status epileptics) rather than to be used as prophylactic anticonvulsant therapy. The reasons behind this difference remains to be established. A likely explanation is, however, an increased availability of extracellular ATP during status epilepticus due to seizure-induced neurodegeneration and/or an increased inflammatory response. The mechanisms how the P2X7R alters seizure severity are still to be determined. It is, however, tempting to speculate that this is mediated, at least in part, by the suppression of inflammatory processes. The P2X7R is highly expressed in microglia under normal control conditions and following status epilepticus (Kaczmarek-Hajek et al. 2018; Morgan et al. 2020) and the P2X7R has been shown to drive microglial activation (Monif et al. 2009). Moreover, blocking of the P2X7R during status epilepticus leads to decreased release of the pro-convulsant cytokine IL-1β in the hippocampus (Vezzani and Baram, 2007; Engel et al. 2012). Although not shown to be increased on astrocytes in experimental models of epilepsy, the P2X7R can also activate astrocytes (Khan et al. 2019), whether directly or indirectly. Astrocytes can reduce the seizure threshold via various mechanism including dysregulation of the extracellular ionic balance, impaired neurotransmitter reuptake, release of pro-inflammatory cytokines and purines (*e.g.,* ATP and adenosine) (Bedner et al. 2015; Robel et al. 2015; Illes et al., 2019a) or an increase in the expression of adenosine kinase (ADK) with the subsequent decreased concentration of adenosine in the extracellular space (Boison, 2008). The P2X7R may, however, also impact on other pathological processes during seizures including changes in neurotransmitter release [*e.g.,* GABA and glutamate as shown by Barros-Barbosa et al. (Barros--Barbosa et al., 2016b)]. Importantly, we still do not know why P2X7R antagonists elicit different responses according to the experimental model used. There are multiple possible explanations (*e.g.,* different models (KA vs. pilocarpine) and P2X7R antagonists used, time-point and dose, species, genetic background, cell-type recruited during seizures/epilepsy etc.). Identifying the reasons behind these differences will be crucial to advance therapies based on the P2X7R towards a clinical application.

The only other P2XR for which a functional role during acute seizures has been described is the P2X4R subtype. Interestingly, the P2X4R is the receptor with which the P2X7R shares most similarities among the P2XR family (Craigie et al. 2013). Using the intraperitoneal KA mouse model, Ulman et al. (Ulmann et al. 2013) found that mice deficient in the P2X4R, despite experiencing no changes in seizure severity during status epilepticus, are partially protected from seizure-induced neuronal cell death possibly via regulating the activation of microglia.

##### 2.4.2.2. The role of P2XRs during epileptogenesis and epilepsy.

While a role for P2XRs during epilepsy has emerged much more recently, we now have substantial data not only regarding changes in their expression but, more importantly, data on the impact of P2XR-trargeting drugs on epileptogenesis and epilepsy. Similar to status epilepticus, this data is mostly restricted to the P2X7R subtype with one study investigating also the role of the P2X3R.

The first evidence for antiepileptogenic effects provided by P2X7R antagonists were reported in a study using the PTZ kindling model in rats, a well-established model of epileptogenesis (Dhir, 2012). Using this model, Soni et al. (Soni et al. 2015) showed that the P2X7R antagonist BBG decreased the mean kindling score and restored cognitive deficits and motor coordination. These findings were confirmed in a more recent study in the same model using the P2X7R antagonists JNJ-47965567, AFC-5128 and, with more modest effects, BBG (Fischer et al. 2016). Further evidence for a pro-epileptogenic role for P2X7R was presented in a study in mice using inhibitors of a P2X7R-suppressing microRNA (microRNA-22). Intra-amygdala KA-injected mice treated with microRNA-22 inhibitors presented increased hippocampal expression of the P2X7R and developed a more severe epileptic phenotype accompanied with increased cytokine release and astrogliosis (Jimenez-Mateos et al. 2015). Using the pilocarpine rat model of status epilepticus, Amorim et al. (Amorim et al. 2017) showed that injection of P2X7R-targeting siRNA 6 h post-status epilepticus not only delayed the emergence of the first seizure, but also reduced the frequency and severity of seizures. In contrast to these findings, treatment with the P2X7R antagonists AZ10606120 and BBG post-pilocarpine-induced status epilepticus in mice resulted in the development of a more severe epileptic phenotype (Rozmer et al. 2017). Evidence for the efficacy of P2X7R antagonists in altering fully established epilepsy is presented by two studies both published in 2016 (Amhaoul et al. 2016; Jimenez-Pacheco et al. 2016). In the first study, Amhaoul et al. (Amhaoul, Ali et al. 2016) used the multiple low-dose i.p. KA model in rats. Epileptic rats treated for one week with the P2X7R antagonist JNJ-47965567, three months following KA-induced status epilepticus, experienced the same number of seizures, however, showed a significant reduction in seizure severity. In the second study, Jimenez-Pacheco et al. (Jimenez-Pacheco et al. 2016) used the intra-amygdala KA mouse model. Mice were treated with the same P2X7R antagonist JNJ-47965567 starting treatment 10-days post-status epilepticus for five days. In contrast to the previous study, P2X7R antagonism reduced the total number of seizures during treatment. Remarkably, this effect persisted during a one-week drug-washout period suggesting disease-modifying potential. Thus, in contrast to acute seizures, the P2X7R seems to play a more prominent role once pathological processes have been initiated. At this stage, inflammatory processes are more likely to play an important role, potentially resulting in increased P2X7R expression and function. In line with P2X7R antagonism suppressing epileptic seizures via blocking inflammation, epileptic mice treated with a P2X7R antagonist (JNJ-47965567) showed a strong reduction in both astrogliosis and microgliosis (Jimenez-Pacheco et al. 2016). While suppressing inflammatory pathways is the most likely explanation, it is, however, important to keep in mind, as mentioned before, that the P2X7R is involved in a myriad of different pathological conditions including BBB disruption, changes in neuro-transmitter levels, synaptic reorganization, neurogenesis to name just a few (Sperlagh and Illes, 2014).

To date, the only other P2XR studied beside the P2X7R during epilepsy is the P2X3R. Here, Xia et al. (Xia et al. 2018) showed by using a PTZ-induced kindling rat model, that the P2X3R antagonist NF110 reduced the mean kindling score and improved other pathological parameters such as memory deficits, motor activity, neuronal damage and hippocampal inflammation.

##### 2.4.2.3. The metabotropic P2YR family as a therapeutic target in status epilepticus and epilepsy.

While among the P2Rs most focus has been put on the study of the fast-acting ionotropic P2XR family, data now also suggests a prominent role for the metabotropic P2YR family during seizures (Alves et al. 2018). These studies are, however, limited to models where status epilepticus was induced via KA or pilocarpine. The first demonstration of a role for P2YRs during status epilepticus stems from a study using the intraperitoneal KA-induced status epilepticus mouse model demonstrating that P2Y_12_R deficiency leads to a suppression of microglial morphological changes associated with status epilepticus and in an exacerbated seizure phenotype (Eyo et al. 2014; Avignone et al. 2015). Further support for a role for P2YRs in regulating microglia activation in epilepsy is presented in a recent study analyzing microglial motility in tissue slices from patients with TLE (Milior et al. 2020). Here, the authors show that while low doses of ADP application triggered microglial process extension, which were blocked via P2Y_12_R antagonists, high doses of ADP caused process retraction and membrane ruffling, blocked by the joint application of P2Y_1_R and P2Y_13_R antagonists. A recent study using a model of intracerebroventricular KA injection has now also shown the P2Y_12_R to be involved in post-status epilepticus-induced neurogenesis (Mo et al. 2019). Most studies, however, focused on the P2Y_1_R subtype. The first *in vivo* evidence demonstrating P2Y_1_R involvement during seizures was published by Simones et al. (Simoes et al. 2018) showing P2Y_1_R antagonism (MRS2179)-mediated neuroprotection following systemic KA and intrahippocampal quinolinic acid in rats. P2Y_1_R antagonism had no impact on seizures in their study. In contrast to these findings, Alves et al. showed in a study published one year later (Alves et al., 2019a), that P2Y_1_R-deficient mice presented a lower seizure threshold, experiencing more severe seizures during intra-amygdala KA-induced status epilepticus with the resulting increase in hippocampal neurodegeneration. In line with a genetic P2Y_1_R deletion, pre-treatment with the specific P2Y_1_R antagonists MRS2500 exacerbated seizure severity and cell death during status epilepticus and, conversely, mice pre-treated with the P2Y_1_R agonist MRS2365 had less severe seizures during status epilepticus and less seizure-induced neurodegeneration. Surprisingly, when the authors used a post-treatment regime, the same P2Y_1_R antagonist suppressed seizures and protected the brain from damage, whereas the P2Y_1_R agonist increased seizure severity, suggesting P2Y_1_R-based treatment to be highly depending on the time-point of intervention. The exact reason for this context-specific role of P2Y_1_R during seizures remains to be elucidated; however, cell-specific changes in the expression of the receptor may represent a possible explanation. In their study, the authors found P2Y_1_R to be mainly expressed on neurons during normal physiological conditions, however, once seizures were provoked and pathological changes initiated within the brain, P2Y_1_R expression increased on microglia. In line with P2Y_1_Rs driving inflammatory processes and thereby contributing to seizures during status epilepticus, treatment with the P2Y_1_R antagonist MRS2500 had no effect in mice pre-treated with the anti-inflammatory drug minocycline (Alves et al., 2019a). Using immunohistochemistry, the authors further showed P2Y_1_R to be undetectable on astrocytes. This is a surprising finding, as other studies have found the P2Y_1_R to be expressed and functional on astrocytes (Franke et al. 2004). The participation of P2Y_1_Rs in astrocyte-astrocyte signaling is well established and there is also evidence of the P2Y_1_R contributing to seizure generation and epilepsy via the mediation of astrocytic-Ca^2+^ oscillations. In line with this, Wellmann et al. showed that, in hippocampal slices from fully kindled rats, targeting the P2Y_1_R decreased the mean duration of astroglial Ca^2+^ oscillations by reducing the frequency of slow Ca^2+^ transients (Wellmann et al. 2018). Nikolic et al. meanwhile, demonstrate that the targeting of P2Y_1_Rs restored normal excitatory synaptic activity in the inflamed hippocampus via the blockade of TNF-α-induced Ca^2+^-dependent glutamate release from astrocytes (Nikolic et al. 2018).

The only study analyzing a functional role for P2YRs during epilepsy was carried out by Alves et al. (Alves et al., 2019a), using the intra-amygdala KA mouse model of status epilepticus. Here, treatment with the P2Y_1_R antagonist MRS2500 post-status epilepticus delayed the onset of epilepsy and treatment during epilepsy suppressed epileptic seizures. Seizure-suppressing effects, however, were transient and did not persist beyond termination of treatment.

**Fig. 2.**
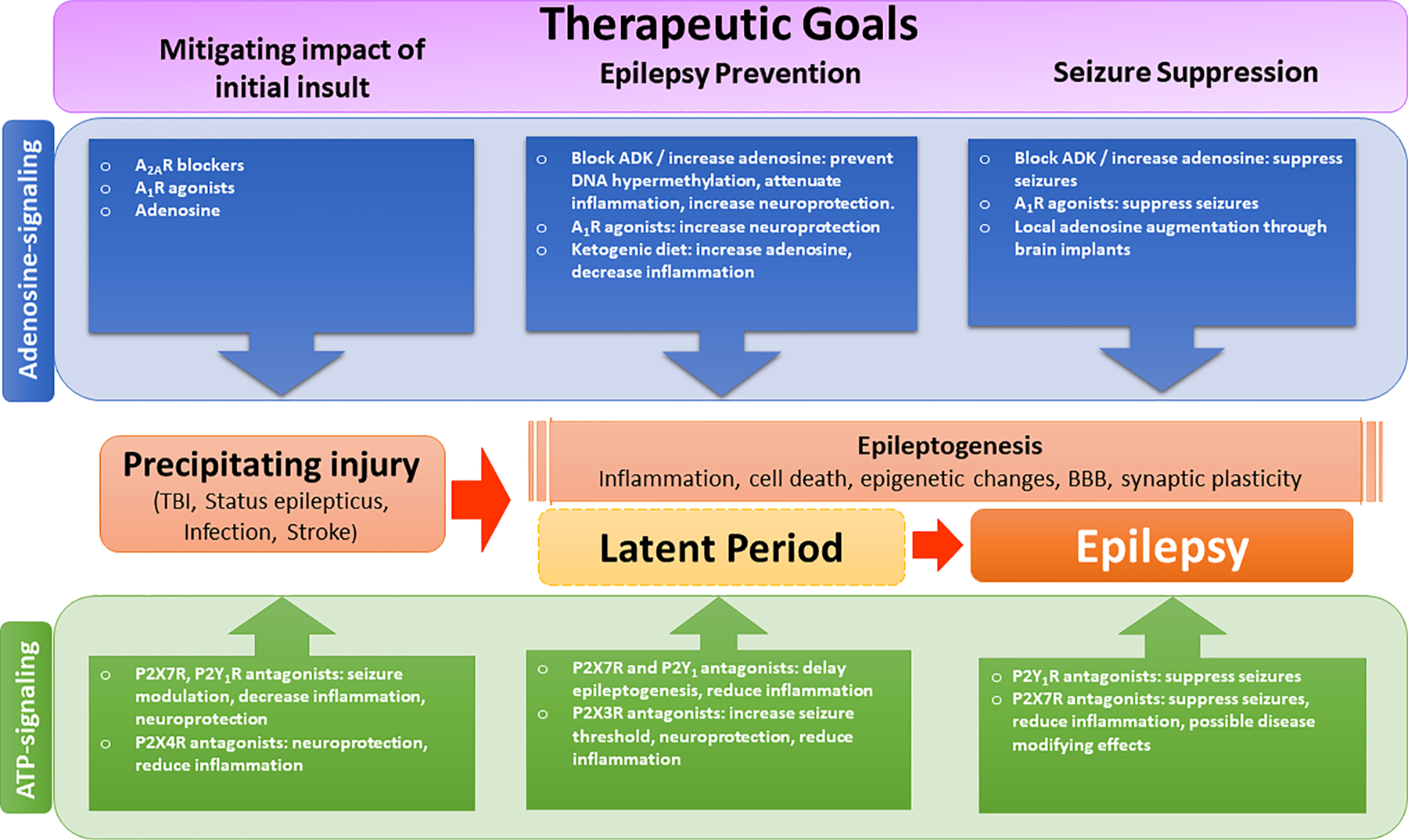
Targeting ATP and adenosine signaling as potential treatment for epilepsy. Epileptogenesis, the development of epilepsy, can be triggered via a precipitating injury to the brain (*e.g.*, traumatic brain injury (TBI), status epilepticus) causing numerous pathological changes (*e.g.*, inflammation, cell death, epigenetic changes) which eventually lead to the occurrence of spontaneous, epileptic seizures. Increasing evidence has demonstrated that ATP and adenosine-dependent signaling plays a key role during seizure generation and the development and maintenance of epilepsy. Targeting specific components of the purinergic signaling cascade can attenuate the precipitating injury, modify the epileptogenic process as such, and finally suppress seizures in epilepsy.

In summary, we have now compelling evidence for a functional contribution not only for P1Rs but also for P2Rs (in particular regarding the P2X7R and P2Y_1_R subtype) (Table 3), to seizure generation, seizureinduced brain damage and development of epilepsy (Fig. 2).

## 3. Purinergic signaling and comorbidities of epilepsy

Targeting of purinergic signaling has been suggested as possible treatment avenue for numerous pathological CNS diseases including the most common comorbidities associated with epilepsy [*e.g.*, depression, schizophrenia (Burnstock, 2008; Illes et al., 2019b; Miras-Portugal et al. 2019)]. According to Feinstein et al. (Feinstein, 1970) comorbidities are defined as “any distinct additional entity that has existed or may occur during the clinical course of a patient who has the index disease (*i.e.* epilepsy) under study.” When referring to epilepsy in terms of comorbidities it is, however, important to keep in mind that epilepsy, rather than constituting a uniform entity, represents a highly heterogeneous condition with differences in etiology, clinical manifestations and treatment responses. Epilepsy, therefore, may be better referred to as a spectrum of disorders featuring increased network excitability resulting in the occurrence of epileptic seizures (Sirven, 2015). It therefore comes to no surprise that epilepsy has a particularly high burden of comorbidities with some conditions up to eight times more common in patients with epilepsy, compared to the general population (Lin et al. 2012). Psychiatric disorders (*e.g.*, mood, anxiety, and psychotic disorders) are one of the most common comorbidities reported in epilepsy and are estimated to occur in 25 %–50 % of patients (LaFrance et al. 2008) and are particularly high in patients with TLE and drug-refractory epilepsy (Bragatti et al. 2010; Patel et al. 2017; Jansen et al. 2019). Other frequently diagnosed comorbidities in epilepsy include migraine, heart diseases, stroke, respiratory diseases (*e.g.*, asthma), allergies and an increased mortality (Keezer et al. 2016). Demonstrating the importance of treating both primary condition (*i.e.*, epilepsy) and associated comorbidities, comorbidities may be linked to poor seizure control (*e.g.*, migraine, psychiatric disorder) or a reduction in the quality of life (*e.g.*, depression) (Velioglu et al. 2005; Taylor et al. 2011). Comorbidities in epilepsy may be caused by direct effects of seizures, adverse effects of ASDs and other treatments or by common pathogenic mechanisms (Keezer et al. 2016), with the latter suggesting that drugs acting on shared pathological processes (*e.g.*, inflammation) may provide the opportunity to target both primary disease and associated comorbidities. On the other hand and further supporting shared pathological pathway activation across different brain diseases, increased brain excitability and seizures are a common comorbidity of numerous other brain diseases including neurodegenerative [e.g., Alzheimer’s disease (Vossel et al. 2016; Lam et al. 2017), Huntington’s disease (Rodrigues and Wild, 2016)] and psychiatric diseases [*e.g.* schizophrenia (Cascella et al. 2009)] where they contribute to disease progression (Vossel et al. 2017; Kanner et al. 2018). Treating patients with non-epileptic diseases with anti-seizure medication may therefore not only reduce hyperexcitability in the brain but also the primary disease pathology. In line with this, ASDs are also in use for the treatment of various non-epileptic CNS conditions including psychiatric disorders (Bialer, 2012).

As mentioned in previous sections, among the P2R family, the ionotropic P2X7R has attracted most attention as possible therapeutic target in diseases of the CNS (Sperlagh and Illes, 2014). An emerging concept is that increased hyperexcitability and network changes are universal patho-mechanisms in numerous chronic brain diseases, caused and maintained by sustained glial activation (Kanner, 2012; Devinsky et al. 2013; Chitnis and Weiner, 2017; Bauer and Teixeira, 2019). The P2X7R has been described as a gatekeeper of inflammation driving the activation of microglia (Monif et al. 2009) whilst also regulating the release of neurotransmitters (*e.g.* glutamate, GABA) (Sperlagh et al. 2002). P2X7R may function, therefore, as a molecular link between glia-driven pro-inflammatory signaling and neuronal hyperexcitability (Henshall and Engel, 2015), thereby contributing to both primary disease pathology and associated comorbidities. In the same line, we recently proposed that a triad of synaptotoxicity, astrogliosis, and adenosine deficiency is a common pathological hallmark shared by epilepsy, Alzheimer’s disease, Parkinson’s disease and amyotrophic lateral sclerosis (Boison and Aronica, 2015). In line with this hypothesis, ADK overexpressing mice with decreased adenosinergic tone show impairment in multiple cognitive domains (Yee et al. 2007), whereas reconstitution of adenosine via the implantation of adenosine releasing cells into the hippocampus improved cognitive function (Shen et al. 2012).

Taken together, targeting of purinergic signaling during epilepsy may not only impact on seizures and the development of epilepsy, but also on associated comorbidities. Likewise, targeting the purinergic signaling system in other CNS diseases may also reduce brain hyperexcitability, the risk of seizures and their impact on disease progression. Despite data demonstrating a role for both ATP and adenosine-mediated signaling in different brain pathologies and diseases, a more systematic approach is needed to investigate how targeting of purinergic signaling impacts on primary disease and its associated comorbidities in the same experimental model.

## 4. Sudden unexpected death in epilepsy (SUDEP) and purinergic signaling

SUDEP remains a major concern for persons with epilepsy and their families. The incidence of SUDEP is estimated at 1–2.5 per 1000 patient years, however the underlying mechanisms are poorly understood. While increased adenosine suppresses neuronal activity as an innate mechanism to stop seizures (Lado and Moshe, 2008), it also suppresses cardiovascular and respiratory functions through increased activation of adenosine receptors in the brainstem (Evans et al. 1982; Barraco and Stefano, 1990; Herlenius et al. 1997). The seizure-induced adenosine surge as discussed above has been associated with the postictal state (Fisher and Schachter, 2000). The ‘adenosine hypothesis of SUDEP’ predicts that a seizure-induced adenosine surge originating from a seizure focus in combination with impaired metabolic clearance of adenosine from the brainstem can trigger lethal suppression of respiratory and/or cardiac functions. There is strong experimental and clinical evidence that the seizure-induced adenosine-surge (even when it originates from one focal area) affects the entire brain, including the brainstem, resulting in a brain-wide ‘post-ictal brain shutdown’ (During and Spencer, 1992; Fedele et al. 2006; Hirsch, 2010; Hesdorffer and Tomson, 2013). If excessive adenosine is a necessary trigger for SUDEP, then AR antagonists such as caffeine, or agents that enhance metabolic clearance of adenosine should prevent SUDEP. Indeed, an acute dose of caffeine was shown to extend life in a lethal seizure model (Shen et al. 2010) and prevent lethal apnea after TBI (Lusardi et al. 2012). These findings suggest that a commonly used psychoactive drug – caffeine – warrants closer attention and scrutiny within the context of epilepsy and SUDEP.

Despite the widespread use of caffeine and related methylxanthines in persons with epilepsy, dietary factors have received little attention in the study of SUDEP. In a study performed at Cleveland Clinic, only 20 % of 301 participants (all candidates for epilepsy surgery) did not consume caffeine, whereas 31 % drank up to 24 ounces of coffee per day, 45 % drank between 24 and 96 ounces, and 3% drank 100 ounces or more per day (Source: Epilepsy.com). It is thus fair to predict that, like the general population, a large proportion of adult persons with epilepsy regularly consume caffeine and related methylxanthines. While the acute use of methylxanthines may increase seizure risk (Boison, 2011), the same stimulants may also reduce risk for SUDEP. In contrast, chronic caffeine use may increase the brain’s vulnerability to SUDEP via upregulation of ARs, which renders the brain more susceptible to the effects of endogenous adenosine, a phenomenon termed ‘effect inversion’ (Jacobson et al. 1996; Fredholm, 1997). The widespread use of caffeine in persons with epilepsy warrants a detailed risk/benefit evaluation of chronic caffeination for SUDEP.

Although the seizure-induced adenosine-surge limits the spatial and temporal extent of a seizure (Fedele et al. 2006; Li et al. 2012), the resulting excess in adenosine needs to be metabolized effectively to avoid excessive postictal depression. As discussed above in the adult brain, the metabolic clearance of adenosine is largely under the control of ADK (Boison et al. 2010; Boison, 2012, 2013). We and others have demonstrated a tight spatial association of overexpressed ADK with epileptogenic brain activity (*e.g.*, in the temporal lobe) (Aronica et al. 2011; Li, Lytle et al. 2012; Aronica et al. 2013). Importantly, over-expression of ADK is restricted to seizure generating brain areas, whereas lower ADK levels outside those areas maintain a protective adenosine tonus, thereby preventing the spread of seizures into adjacent or remote brain areas (Fedele et al. 2006; Li et al. 2012). Thus, reduced ADK expression in the brainstem would prevent seizure spread into the brainstem, but would, at the same time, increase SUDEP risk. Three lines of evidence support a critical role for adenosine and defects in its metabolic clearance for the development of lethal apnea: *(i)* A combination of a seizure with pharmacological disruption of metabolic adenosine clearance triggered death in normal mice (Shen et al. 2010). Conversely, a single acute dose of caffeine attenuated this outcome. *(ii)* Like seizures, severe TBI is associated with a surge in adenosine, and high CSF levels of adenosine correlated with lethal outcomes in human subjects (Clark et al. 1997). On the other hand, an acute bolus of caffeine given immediately after severe TBI in rats prevented apnea and mortality (Lusardi et al. 2012). *(iii)* SUDEP may share a similar etiology with sudden infant death syndrome (SIDS) (Hirsch, 2010; Tao et al. 2010), a condition that has been linked to global ADK deficiency in neonates (Boison et al. 2002). Deficiencies in the metabolic clearance of adenosine, therefore, constitute a candidate mechanism to trigger SUDEP.

## 5. Extracellular purine metabolism

### 5.1. Ectoenzymes

Changes in extracellular purine concentrations are not only dependent on the rate of release from cellular sources, but also on the rate of cellular reuptake (discussed in section 5.3) and of extracellular metabolism. With ATP and adenosine often acting as ligands for receptors mediating mutually antagonistic signalling pathways (Yegutkin et al. 2006), the rate at which ATP is broken down into adenosine in the extracellular space has a double impact: a decrease in the efficiency of ATP breakdown will lead, not only to excessive activation of P2 receptors, but also a reduced activity of P1-activated pathways – the ratio of extracellular purines is of critical importance and largely mediated by the activity of ectoenzymes (Zimmermann, 2000). ATP is metabolized to adenosine, often through a cascade of enzymes, with intermediate purine products, such as ADP and AMP also functioning as ligands for certain P2 receptors (discussed in section 2.2). ATP is a substrate for four principal families of ectoenzymes: the ectonucleoside triphosphate diphosphohydrolase family (E-NTPDases), the ectonucleotide pyrophosphatase/phosphodiesterase family (E-NNP), and the alkaline phosphatases (ALC) (Yegutkin et al. 2006).

Of the E-NTPDases, E-NTPDase 1 (CD39), 2, 3, and 8 are expressed on the extracellular cell surface, with CD39 both the most highly expressed and most extensively characterized (Robson et al., 2006). All the above E-NTPDases metabolize adenine and to a lesser extent, uridine nucleotides, with varying affinities. While CD39 has approximately equal affinity for ATP and ADP, E-NTPDase 2 is almost completely selective for ATP. E-NTPDase 3 and 8, meanwhile, have a higher affinity for ATP than ADP, but are less selective than E-NTPDase 2 (Robson, Sevigny et al. 2006). The E-NTPDase family all catalyse the degradation of ATP or ADP into AMP (or UTP or UDP to UMP) (Schetinger et al. 2007). Similarly, members of the E-NNP family cleave ATP to AMP (Masse et al. 2010). As well as acting as a ligand at certain P2 receptors, AMP forms the substrate for the ecto-5’nucleotidase (CD73)-dependent catalysis of adenosine (Strater, 2006). ALC enzymes, meanwhile, provide a rather non-selective cleavage route for the conversion of ATP into adenosine (Huizinga et al. 2012). The sequential action of CD39 and CD73 however is the major extracellular pathway responsible for the cleavage of ATP into adenosine (Cunha et al. 1992; Zimmermann, 2000; Koszalka et al. 2004; Fredholm et al., 2011; Augusto et al. 2013; Chu et al. 2014; Borea et al. 2018; Boison and Yegutkin, 2019).

While the concentration and activity of ectonucleotidases plays a role in determining the ratio of extracellular ATP to its metabolites in the extracellular space, with adenosine also released directly from the intracellular compartment, the extent to which the extracellular pool of ATP contributes to adenosine is not fully clear. While a study by Langer et al. (Langer et al. 2008) demonstrated that dephosphorylation of AMP to adenosine is largely restricted to the striatum and olfactory bulb, other studies have demonstrated CD73-dependent ATP metabolism to adenosine (Klyuch et al. 2012) in the cerebellum (Wall et al. 2007) and hippocampus (Wall and Dale, 2013). Experiments carried out on astrocyte/neuron co-cultures from CD73 KO mice, or with the use of CD73 inhibitors suggest that this enzyme contributes significantly to extracellular adenosine pools (Chu et al. 2014). Interestingly, CD73 is inhibited by ATP and ADP, allowing for the spatial and temporal separation of ATP release sites and sites of adenosine production (Dale, 1998, 2002; Masse and Dale, 2012).

Reduced activity of ectonucleotidases in genetic mouse models lowers the seizure threshold (Lanser et al. 2017), while following experimental status epilepticus, a substantial increase in CD73 in the hippocampus was observed in response to systemic pilocarpine or KA in –rats (Bonan et al. 2000). These results are consistent with human studies, in which CD73 was elevated in the hippocampus of TLE patients (Lie et al. 1999; Barros-Barbosa et al., 2016a). Further, genetic variations in CD73 are associated with greater risk of epilepsy development following TBI (Diamond et al. 2015). Theoretically, increasing the activity of CD73 might be a potential anti-epileptic treatment strategy (Schetinger et al. 2007; Sebastian-Serrano et al. 2015), however, pharmacological activators are challenging to design leaving gene therapy to overexpress CD73 as a potential option. Further, recent data from proximity ligation assays demonstrates a physical association between CD73 and excitatory A_2A_Rs, implying functional coupling (Barros-Barbosa et al., 2016a). This suggests that activation of CD73 may have an excitatory effect.

### 5.2. Adenosine transporters

Extracellular adenosine is transported into the cell by the SLC29 family of equilibrative nucleoside transporters (ENTs) and concentrative nucleoside transporters (CNTs). The four ENT (1–4) isoforms are highly expressed throughout mammalian tissues with differences in their abundance (Table 4). ENT1 and ENT2 are highly expressed in the brain (Baldwin et al. 2004), whereas the pH-dependent transporters ENT3 and ENT4 are expressed in intracellular membranes and cardiac tissue, respectively (Baldwin et al. 2005; Barnes et al. 2006). In contrast to the wide expression of ENTs, the expression of CNTs is more limited (Jennings et al. 2001). Depending on the adenosine concentration, the four ENT (1–4) isoforms play a dynamic role in intra- and extracellular adenosine homeostasis, transporting nucleosides into or out of the cell. Simply stated, if intracellular adenosine levels are compromised, there is an influx of adenosine from the extracellular space through ENTs and vice versa (Boison, 2013). For this reason, ENT1 blockers such as dipyridamole increase the availability of extracellular adenosine as mechanism of action for their anticonvulsant effects (Kong et al. 2004; Carrier et al. 2006; Boison, 2008; Cieslak et al. 2017). The role of the three CNT (1–3) isoforms is to regulate the transport of adenosine using sodium ion gradients as a source of energy (Borea et al. 2018). Among the three nucleoside transporters, CNT2 is the major transporter of adenosine. Interestingly CNT2 activity is controlled by A_1_Rs and therefore contributes to the regulation of A_1_R signaling through an internal feedback loop (Aymerich et al. 2005). While the implications of CNTs in epilepsy are not yet established, the termination of extracellular adenosine signaling requires the synergistic action of both ENT and CNT

### 5.3. Intracellular adenosine metabolism

Adenosine is well known for its inhibitory effects in the brain and a large body of evidence is available regarding its receptor-dependent endogenous anticonvulsant effects (Boison, 2016). Intracellularly, adenosine mainly originates from the hydrolysis of S-adenosyl-L-homocysteine (SAH) and is metabolized by either ADK or adenosine deaminase (ADA). Between those two enzymes, ADK, as a low-capacity, low-K_m_ enzyme, is the key metabolic regulator of intracellular adenosine under basal conditions (Boison, 2010). In the adult brain, both isoforms of ADK (nuclear ADK-L and cytoplasmic ADK-S) are predominantly expressed in astrocytes (Studer et al., 2006; Boison, 2013). Due to the existence of the ubiquitous equilibrative transporters for adenosine, a reduction in intracellular ADK activity increases both intra- and extracellular adenosine levels and consequently activation of A_1_Rs. In the following, “ADK” applies to both isoforms if not detailed otherwise. ADK-L has recently been identified as an epigenetic regulator of DNA methylation, an important new role, discussed below, implicated in epileptogenesis, neurogenesis, cell proliferation, and cancer (Williams-Karnesky et al. 2013; Boison and Rho, 2019; Gebril et al., 2020). In line with a role in the regulation of cell proliferation and plasticity, ADK-L is widely expressed in developing neurons (Studer et al., 2006) and its expression in adulthood is only maintained in the nuclei of dentate granule cells (Gebril et al., 2020) and granular neurons and Purkinje cells of the cerebellum (unpublished data). Cytoplasmic ADK-S, meanwhile, regulates the tissue tone of adenosine and AR activation (Boison, 2013). The two isoforms not only vary in their function, but also in their levels of expression at different developmental stages. During early postnatal brain development, under physiological conditions, there is a developmental shift in hippocampal ADK gene expression from predominantly neuronal at P4 to predominantly astrocytic at P14, as well as a shift in specific isoform expression where ADK-L is down-regulated in neurons and ADK-S upregulated in astrocytes during the same time window. While the exact mechanisms involved in these changes in transcription are unclear, it is possible that, as previously described for the P2X7R (Engel et al. 2017), these changes are also regulated by the transcription factor SP1, because of its binding to the ADK-L promoter in neurons at P4 (Kiese et al. 2016).

### 5.4. Adenosine receptor-independent, epigenetic antiepileptogenic effects of adenosine

Seizures can induce epigenetic alterations and thereby aggravate the epileptogenic condition (Kobow and Blumcke, 2011). Specifically, increased activity of DNA methylating enzymes and hypermethylated DNA has been associated with the development of human and experimental epilepsy (Kobow et al. 2009; Zhu et al. 2012; Kobow et al. 2013; Williams-Karnesky et al. 2013; Miller-Delaney et al. 2015). Interference with DNA methylation therefore offers novel opportunities for epilepsy prevention.

We recently discovered a novel epigenetic function of adenosine in epilepsy, where adenosine assumes a key role as regulator of DNA methylation (Williams-Karnesky et al. 2013). DNA methylation requires the donation of a methyl group from S-adenosylmethionine (SAM), catalyzed by DNA methyltransferases (DNMTs). The resulting product, S-adenosylhomocysteine (SAH) is converted into adenosine and homocysteine (HCY) by SAH hydrolase. This reaction will only proceed when adenosine and HCY are constantly removed (Kredich and Martin, 1977; Boison et al. 2002). If metabolic clearance of adenosine through ADK is impaired, SAH levels rise (Boison et al. 2002). SAH in turn inhibits DNMTs through product inhibition (James et al. 2002). Based on adenosine’s role as a product of DNA methylation, an increase in ADK and the resulting decrease in adenosine, as seen in epilepsy (Li et al. 2008; Masino et al. 2011), drives increased global DNA methylation in the brain. Adenosine augmentation reverses pathological DNA hypermethylation and thereby prevents epilepsy progression.

The antiepileptogenic potential of transient focal adenosine-delivery was directly tested in a rat model of systemic KA-induced progressive TLE (Williams-Karnesky et al. 2013). Young male rats received an acute dose of systemic KA to trigger acute convulsive status epilepticus. Once reaching a stable rate of 3–4 spontaneous recurrent seizures per week at 9 weeks post-KA, the rats were randomized and each received bilateral intraventricular adenosine-releasing silk-implants, silk-only implants, or a corresponding sham treatment. Adenosine implants were designed to transiently deliver a stable dose of 250 ng adenosine / ventricle / day restricted to 10 days of drug delivery (Szybala et al. 2009). 24 h post-surgery, continuous video monitoring was initiated, maintained for 4 weeks, and resumed after a 4 week hiatus for an additional 4 weeks. In the control groups seizures continued to increase both in number and severity. In contrast, adenosine-releasing implants almost completely suppressed seizures. Remarkably, reduced seizure activity was maintained far beyond expiration of adenosine-release from the polymer and at least 12 weeks post implantation. Even 12 weeks post implantation, seizure incidence was reduced by ≥ 70 %. In line with sustained seizure reduction, mossy fiber sprouting at 21 weeks post-KA was significantly attenuated in adenosine-treated rats. Importantly, transient delivery of adenosine restored normal DNA methylation long-term and epilepsy progression was halted.

## 6. Adenosine augmentation therapies

The anticonvulsant, antiepileptogenic and neuroprotective properties of adenosine are well-established (Boison, 2016). This section focuses on approaches to augment adenosine in the brain, and thereby increase the seizure threshold, prevent the emergence of epilepsy or arrest disease progression.

### 6.1. ADK inhibitors

The ADK hypothesis of epileptogenesis formulated in 2008 (Boison, 2008; Li et al. 2008) states that maladaptive overexpression of ADK is intrinsically linked to epileptogenesis and disease progression. It has been demonstrated that overexpression of ADK drives epileptogenesis through enhancing the rate of DNA-methylation (Williams-Karnesky et al. 2013). Because ADK progressively increases in line with epilepsy development and progression (Li et al., 2007a), and because epileptogenic activity originates from ADK-expressing brain areas (Li et al. 2008; Li et al. 2012), ADK is a biomarker to identify epileptogenic brain areas and to monitor epileptogenesis longitudinally. At the same time, ADK is a rational target for therapeutic intervention.

The first antiepileptogenic effect of ADK inhibitors was demonstrated by Sandau et al. in 2019 (Sandau et al. 2019) by showing that the transient use of the non-selective ADK inhibitor 5-ITU has potent antiepileptogenic effects in the intrahippocampal KA model of TLE. Three days after intrahippocampal KA-induced status epilepticus in mice, 5-ITU (1.6 mg/kg i.p. bid) or vehicle was injected for a restricted time span of 5 days. Six weeks after status epilepticus, hippocampal paroxysmal discharges (HPD) were quantified by intrahippocampal EEGs and brains were processed for histopathology. Most animals (16/17) from the KA/vehicle-injected control group developed a robust epilepsy phenotype by 6 weeks after the status epilepticus (23.5 ± 2.1 seizures / hour). In marked contrast, 59 % of all KA/5-ITU treated mice (10/17) were characterized with >95 % reduction of seizure rate and time spent in seizure. Importantly, 3 mice were completely seizure free. Histopathological analysis demonstrated characteristic granule cell dispersion in KA/vehicle-injected control animals that was prevented in KA/5-ITU-protected animals. These data demonstrate a potent anti-epileptogenic effect of the non-selective ADK-inhibitor 5-ITU given transiently during the latent period of epileptogenesis. Importantly, >95 % reduction of seizures in the majority of KA-injected animals was maintained 5 weeks after drug washout, indicating a lasting anti-epileptogenic effect.

ADK inhibitors can produce two distinct effects: *(i)* Blockade of cytoplasmic ADK-S raises extracellular adenosine, and thereby provides enhanced AR activation. Through this enhanced activation of A_1_Rs, ADK inhibitors suppress epileptic seizures (Wiesner et al. 1999; Gouder et al. 2004; McGaraughty et al. 2005), a focus of past drug development efforts. *(ii)* Blockade of nuclear ADK-L increases adenosine in the nucleus and thereby exerts epigenetic effects that are antiepileptogenic (Williams-Karnesky et al. 2013). In the past, differences between ADK-S and ADK-L had not been considered in drug development approaches. Because ADK-L has different (nuclear) protein interaction partners as compared to cytoplasmic ADK-S, there is a solid rationale to develop novel ADK inhibitors with enhanced epigenetic activity, mediated by ADK-L inhibition. In conclusion ADK inhibitors combine epigenetic with antiepileptogenic activity. ADK is a validated target for the prevention of epileptogenesis, which provides the premise to target ADK-L linked epigenetic alterations for antiepileptogenic therapy.

### 6.2. Gene and cell therapy

Systemic adenosine augmentation is associated with receptor-mediated, mostly cardiovascular, side effects. In an attempt to bypass adverse events of systemic adenosine augmentation, focal adenosine augmentation therapies were tested experimentally to enhance adenosine concentrations locally by using gene and cell therapy approaches. For this reason, local gene therapy designed to increase endogenous adenosine by inhibiting or knocking out the *ADK* gene, and restricted to an epileptogenic brain area with pathological overexpression of ADK, stands as a promising strategy for achieving seizure control in refractory epilepsy, with fewer or less severe side-effects (Huber et al. 2001; Guttinger et al. 2005). In line with this therapeutic rationale, injection of an astrocyte-specific adeno-associated virus vector (AVV), which expressed an *Adk* cDNA in antisense orientation into the CA3 subfield of the hippocampus of epileptic *Adk-tg* mice, reduced the seizure rate from 5.8 to 0.6 seizures per hour. Importantly, a gene therapy-induced decrease of only 3.1 % in ADK expression levels was found to be sufficient for seizure reduction (Lee et al. 2008; Boison, 2010; Theofilas et al. 2011). An example of a local cell therapy approach to treat seizures in modelled epilepsy is based on lentiviral RNAi-mediated downregulation of ADK. To achieve this, human mesenchymal stem cells (HMSC) were infected with a lentivirus that expresses an anti-*Adk* microRNA, which led to an 80 % decrease in ADK expression and an 8.5 ng/mL increase in adenosine levels in supernatants of the cultured cells. These HMSC cells were then transplanted into the mouse hippocampus one week prior to intra-amygdaloid KA injections, which in turn led to a 35 % decrease in seizure duration and 65 % decrease in seizure-induced hippocampal neuronal cell loss (Ren et al., 2007; Li et al. 2009). An alternative approach is the use of embryonic stem cells engineered to release adenosine by genetic disruption of ADK (*Adk-/-* cells), which can be differentiated into neural precursor cells prior to grafting into the intra-hippocampal fissures of rats and mice. This approach was shown to suppress epileptogenesis and seizure progression in kindled rats (Li et al., 2007b) and was demonstrated to prevent epileptogenesis in the mouse intra-amygdaloid KA model (Li et al. 2008). Together, those studies demonstrate the potential of focal adenosine augmentation therapies to suppress seizures in epilepsy, and to prevent the development of epilepsy.

### 6.3. Adenosine augmentation via ketogenic diet (KD)

The high fat, low carbohydrate ketogenic diet (KD), where 90 % of energy is derived from fats and 10 % from proteins and carbohydrates combined, has been used for almost 100 years for the treatment of drug resistant epilepsy, specifically in children (Freeman et al. 2009; Kossoff and Rho, 2009; Stafstrom and Rho, 2012). A systematic review on KD from 2018 outlined data from eleven randomized controlled trails including 712 children and 66 adult patients. In this review, an 85 % seizure reduction rate was observed at three months after initiation of KD therapy (Martin-McGill et al. 2018). In an attempt to make KD more tolerable and compliant, variations of KD such as modified Atkins diet (MAD), that includes daily calorie intake of 10 % carbohydrates with 60 % of fats and 30 % of protein’s was proposed (Cervenka et al. 2016). In a recent clinical study, adult patients, when treated for 24 weeks with a MAD, showed profound seizure reduction over a period of 12 weeks (Cervenka et al. 2016; de Souza Neves et al., 2020). The reason for the success of KD therapy is likely based on the fact that KD therapy combines several beneficial mechanisms, which include glucose restriction, free fatty acids and ketone bodies (Bough and Rho 2007). In addition, KD increases adenosine levels through: *(i)* Increases in acetyl-CoA which enhances mitochondrial ATP production and thereby adenosine production via ATP-degrading enzymes (Masino and Geiger, 2008, 2009). *(ii)* Reduced extracellular glucose which induces autocrine regulation of hippocampal CA3 neurons leading to the release of ATP and the consequent increase in extracellular adenosine concentration and activation of ATP-sensitive K^+^ channels (K_ATP_) (Kawamura et al. 2010). *(iii)* Downregulation of ADK expression, an effect that is thought to be triggered via KD-induced metabolic stress (Masino et al. 2011; Lusardi et al. 2015). The KD-induced increase in adenosine levels from all three mechanisms leads to increased activation of A_1_Rs, mediating robust anticonvulsant effects (Masino et al. 2011; Masino et al. 2012).

An exciting new role of KD therapy is suppression of epileptogenesis and disease progression, which was demonstrated in two different rodent models of epilepsy, PTZ-kindled mice and pilocarpine-induced epilepsy in rats (Lusardi et al. 2015). This study demonstrated lasting antiepileptogenic effects even after diet reversal to a control diet. The observed antiepileptogenic effects were associated with the KD’s ability to restore normal adenosine levels in the epileptic brain and to reverse DNA hypermethylation (Lusardi et al. 2015). Taken together, those data demonstrate that adenosine contributes to the potent anticonvulsant, anti-epileptogenic, and disease-modifying properties of KD-based therapy.

## 7. Summary and outlook

**Fig. 3.**
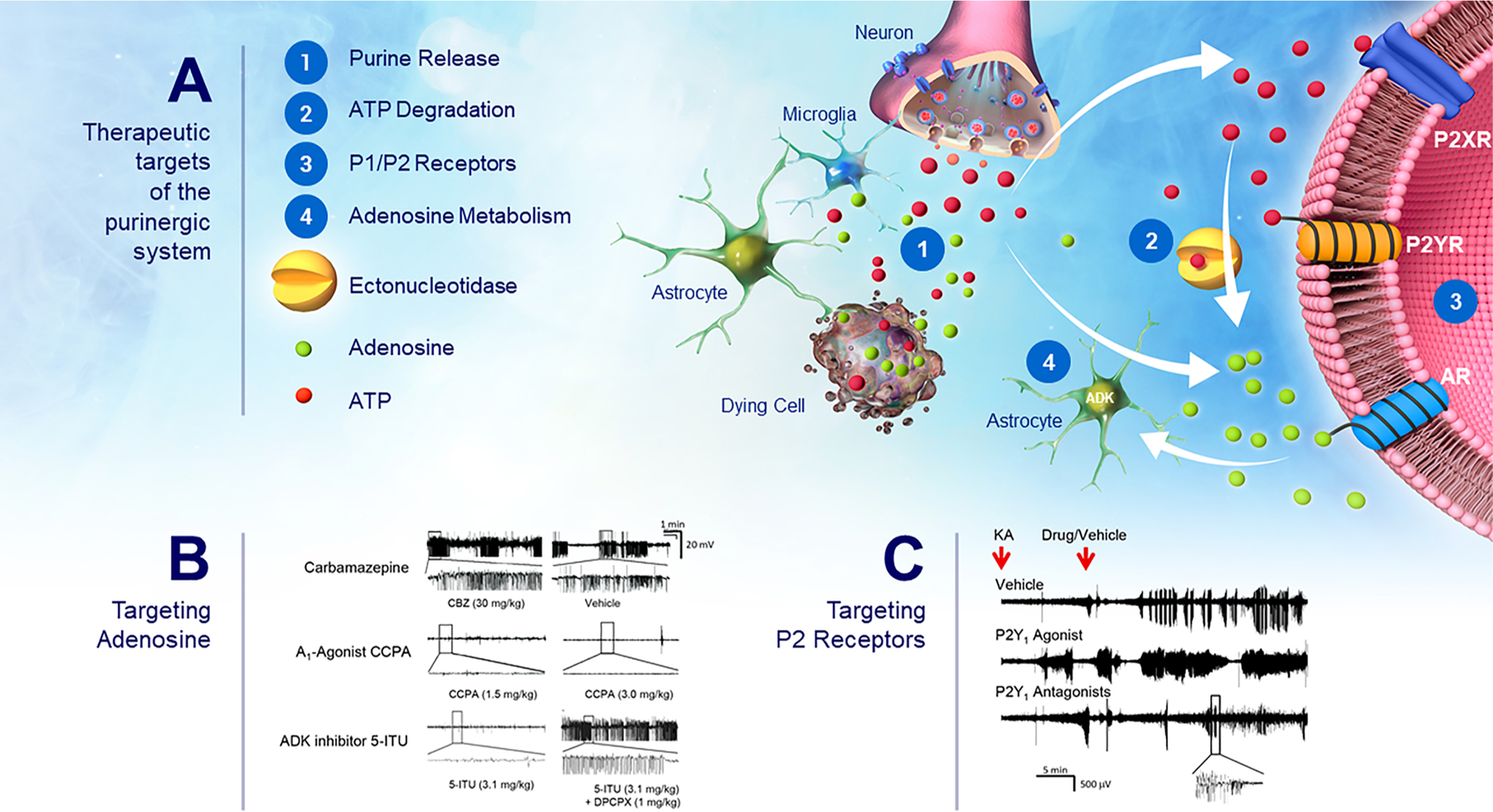
Targeting of the purinergic system to treat epilepsy. (A) Schematic illustrating possible intervention points targeting different components of the purinergic system to suppress seizures and to treat epilepsy. This includes: *(1)* Purine release mechanisms: Purines including ATP and adenosine are released in the brain from neurons and glial cells via active release mechanisms (*e.g.,* via synaptic vesicles, pannexin-1 and connexin-43 hemichannels) or from dying cells. *(2)* ATP-metabolizing ectonucleotidases: Once release into the extracellular space, ATP is rapidly degraded into different breakdown products such as ADP, AMP and adenosine which often mediate opposing effects via their different receptors including P1 and P2 receptors. *(3)* Purinergic receptors: This includes P1 receptors (A_1_, A_2A_, A_2B_, A_3_) activated by extracellular adenosine and P2 receptors which are activated by extracellular adenine and uridine nucleotides (e.g., ATP) and are further subdivided into the ionotropic P2X receptor family and the metabotropic P2Y receptor family. *(4)* Adenosine kinase (ADK): ADK which is predominantly expressed in astrocytes catalyzes the phosphorylation of adenosine to AMP thereby removing extracellular adenosine. (B) Representative EEG traces depicting high amplitude high frequency spiking during intra-amygdala KA-induced status epilepticus. While treatment with the anti-seizure drug Carbamazepine had no effect on seizure severity during status epilepticus, the A_1_R agonist CCPA and the ADK inhibitor 5-ITU suppressed seizure activity. The seizure-suppressive effects provided via 5-ITU were reversed by treatment with DPCPX (A_1_R antagonists). (C) EEG traces during intra-amygdala KA-induced status epilepticus showing increased seizure severity when mice were treated with the P2Y_1_ agonist MRS2500 and seizure suppression when treated with the P2Y_1_ antagonists MRS2365. Each trace is from a different animal. Drugs/Vehicle was administered 15 min post-intra-amygdala KA injection.

There is now compelling evidence demonstrating the therapeutic potential of targeting the purinergic system during acute seizures, the development of epilepsy, and during epilepsy. This includes targeting of different receptor subtypes (including P1 and P2 receptors) and mechanisms of purine release and metabolism (Fig. 3). Of particular promise are the disease-modifying effects observed via targeting of the ATP-gated P2X7R or ADK (Jimenez-Pacheco et al. 2016; Sandau et al. 2019). The observed disease-modifying effects are even more remarkable as treatment of epileptic animals with anti-epileptic or anti-inflammatory drugs suppressed seizures during treatment but not during the wash-out period (Grabenstatter et al. 2007; Maroso et al. 2010; Klein et al. 2015). To date, approaches to identify drug targets based on purinergic signaling are restricted to the targeting of one specific pathway/receptor. The purinergic system is, however, a complex signaling system with one component affecting multiple targets. For example, inhibition of ATP release may prevent activation of pro-convulsive P2 receptors. Decreased ATP may, however, also lead to a decrease in extracellular concentrations of anticonvulsive adenosine. The targeting of several components of the purinergic system simultaneously may therefore be a better option, taking into account different aspects of the purinergic cascade. To take full advantage of the therapeutic possibilities provided by the purinergic system to treat epilepsy, several questions must be resolved before findings can be advanced beyond a preclinical stage.

- Most findings to date are based on rodent models of TLE, but it will be important to investigate whether mechanisms implicated in TLE and its development are also applicable to other forms of epilepsy and at different developmental stages. For example, P2X7R antagonism has been shown to be anti-convulsive in mouse pups subjected to hypoxia-induced seizures (Rodriguez-Alvarez et al. 2017) and rat pups treated with intra-amygdala KA (Mesuret et al. 2014) suggesting anticonvulsive effects are independent on developmental stage (Menendez Mendez et al. 2020).
- While slice work has shown increased extracellular ATP and adenosine concentrations during seizures, our understanding of the *in vivo* dynamics is limited. What are the mechanisms contributing to increased ATP release, and can these be targeted? What ectonucleotidases contribute to ATP degradation and what are the effects of targeting these enzymes? What about uridine nucleotides (*e.g.,* UTP)? Are these also increased following seizures?
- What receptors are recruited during seizures and epilepsy? Compelling evidence has demonstrated a role for the P2X7R and the P2Y_1_R and to some extent for the P2X4R and the P2Y_12_R. Do other P2 receptors also contribute to seizures and epilepsy development? While the contribution of A_1_R and A_2A_R to epilepsy seems to be well-established, the roles for A_2B_R and A_3_R remain incompletely understood.
- For the majority of receptors we have now data showing expressional changes in different brain structures following acute seizures and during epilepsy (including data from patients). However, we still do not know the precise cell types (*e.g.,* glia vs. neurons) and subcellular compartment (*e.g.,* pre- and/or postsynaptic). While reporter mice exist for some of the receptors and highly specific nanobodies are developed [*e.g.,* P2X7R and P2X4R (Kaczmarek-Hajek et al. 2018; Bergmann et al. 2019)], reliable detection tools are still missing for most of the purinergic receptors.
- Purinergic receptors may elicit a different response according to cell type targeted [*e.g.,* neuronal P2Y_1_R expression seems to be anticonvulsive whereas P2Y_1_R expressed on microglia may be proconvulsive (Alves et al., 2019a)]. Thus, cell type-specific KO/overexpression approaches should be undertaken.
- Purinergic signaling has mainly been implicated in mediating inflammatory responses, in particular via P2 receptors. P1 and P2 signaling, however, also affects many more cellular process, therefore, future studies should establish signaling downstream of purinergic receptors during seizures and epilepsy.
- To date, the majority of studies analyzing the role of purinergic signaling in epilepsy have been mainly limited to rodent models of acute seizures (*e.g.*, PTZ, maximal electroshock) or models mimicking TLE (*e.g.*, KA- or pilocarpine-induced status epilepticus) which has provided encouraging data. Epilepsy encompasses, however, a heterogeneous group of brain disorders with many different etiologies (*e.g.*, brain damage, mutations) and underlying pathologies. Thus, to obtain a more complete picture of purinergic signaling during seizure-related disorders more models must be tested (*e.g.*, genetic models) and, in the case of TLE, animal models should also be used which mimic closer the human condition [*e.g.,* TBI model of epilepsy (Pitkanen et al. 2009)].
- Are drugs targeting the purinergic system safe? Brain-penetrant P2X7R antagonists have recently been proven to be safe during clinical trials (Timmers et al. 2018); however, we do not know the effects of purinergic signaling-based long-term treatments, required for chronic brain diseases such as epilepsy. Purinergic receptors are expressed throughout the body (Burnstock, 2008). Are strategies targeting specifically brain cells better (*e.g.,* viral delivery) than the simple administration of drugs available throughout the body?
- The role of sex differences are increasingly recognized in brain functions and diseases of the CNS, including epilepsy (Christian et al. 2020). In general, epilepsy tends to be slightly more common in males than females. While the reasons for these differences are still a matter of debate, several potential mechanisms have been proposed such as differences in brain development (Ruigrok et al. 2014), hormonal and neurosteroidal levels (Chen et al. 2018) and glial function including astrocytes and microglia (Schwarz and Bilbo, 2012). Further demonstrating sex differences impacting on seizures and epilepsy, several models of epilepsy show differences in responses to pro-convulsant stimuli [*e.g.*, male mice show reduced survival and greater seizure severity, cell loss and gliosis in the intrahippocampal KA mouse model of TLE (Li and Liu, 2019)]. Purine receptors have been shown to be expressed differentially according to sex (Crain et al. 2009). Importantly, data has shown dependency on sex of animals on treatments based on purinergic signaling previously [*e.g*., P2X7R antagonism was more effective in female mice in a mouse models of amyotrophic lateral sclerosis (Bartlett et al. 2017)]. While mixed sex groups have been used in some studies [*e.g.*, (Eyo et al. 2014; Rozmer et al. 2017)] to our knowledge, whether targeting of the purinergic system during epilepsy leads to differences according to sex of the animals has not been addressed in a systematic manner to date (see also Tables 2 and 3) and should be investigated in the future.
- Importantly, as mentioned previously, to take full advantage of the purinergic system, multi-targeting approaches should be carried out (*e.g.,* activate A_1_Rs and at the same time block P2X7Rs).
- While prior research efforts have focused on the anti-seizure mechanisms of purines, novel findings demonstrate a key role of adenosine-mediated epigenetic mechanisms in the epileptogenic process itself, and open up a new frontier for the development of novel disease-modifying therapeutics.
- Finally, besides representing exciting therapeutic targets, the purinergic system provides also opportunities for biomarkers development. Blood-based inflammation markers may have a very strong diagnostic and prognostic potential in epilepsy (Vezzani et al. 2019). Purines are released in the brain and can be detected in the blood. Demonstrating the diagnostic potential of blood purine concentration changes, blood purine levels correlate closely with seizure severity during status epilepticus in mice and are elevated in patients with epilepsy (Beamer et al. 2021).

In summary, while there are still unanswered question which must be addressed in the future, we now have a substantial body of work demonstrating the therapeutic potential of targeting the purinergic system during epilepsy and its development.

## Figures and Tables

**Table 1:** *In vivo* approaches employed to investigate purinergic signaling during seizures and epilepsy using adult rodents (selected examples).

Animal model	Type of Seizures / Epilepsy	Induction	Studies on purinergic signaling
PTZ seizure model	Acute generalized seizures	Intraperitoneal, intravenous, or subcutaneous injection of PTZ	P2X7R (mice) ( Fischer et al. 2016); P2X7R (mice) (Nieoczym et al. 2017)
PTZ kindling model	Partial seizures developing into secondarily generalized seizures	Continuous low-dose intraperitoneal administration of PTZ	A_2A_R (mice) (El Yacoubi et al. 2009); P2X7R (species not specified) (Soni et al. 2015); P2X7R (rats, mice) (Fischer et al. 2016); P2X3R (rats) (Xia et al. 2018)
Pilocarpine model of status epilepticus	Status epilepticus, TLE with hippocampal sclerosis	Intraperitoneal injection of pilocarpine	A_2A_R (mice) (El Yacoubi et al. 2009); P2X7R (rats) (Kim et al. 2009; Kim et al. 2010, 2011); P2X7R (mice) ( Kim and Kang, 2011); P2X7R (rats) (Amhaoul et al. 2016); A_1_R (rats) (Amorim et al. 2016); ATP release (rats) ( Dona et al. 2016; Lietsche et al. 2016); P2X7R (rats) (Rozmer et al. 2017)
Intraperitoneal KA model of status epilepticus	Status epilepticus, TLE with hippocampal sclerosis	Intraperitoneal injection of KA	P2X7R (mice) ( Kim and Kang, 2011); P2X4R (mice) (Ulmann et al. 2013); P2Y_12_R (mice) ( Eyo et al. 2014); P2X7R (rats) ( Amhaoul et al. 2016); P2Y_1_R (rats) (Simoes et al. 2018)
Intrahippocampal KA model of status epilepticus	Status epilepticus, TLE with hippocampal sclerosis	Intrahippocampal injection of KA	A_1_R (mice) ( Gouder et al. 2003; Fedele et al. 2006); ADK (mice) (Gouder et al. 2004; Fedele et al. 2005; Sandau et al. 2019); A_1_R (rats) ( Canas et al. 2018)
Intraamygdala KA model of status epilepticus	Status epilepticus, TLE with hippocampal sclerosis	Intraamygdala injection of KA	P2X7R (mice) ( Engel et al. 2012; Jimenez-Pacheco et al. 2013; Jimenez-Mateos et al. 2015, Jimenez-Pacheco et al. 2016); P2YR family (mice) ( Alves et al. 2017; Alves, De et al. 2019; Alves et al. 2019)
Controlled cortical impact (CCI)	Post-traumatic epilepsy, epileptogenesis, hippocampal sclerosis	Mechanical energy delivered to an intact dura exposed following a craniectomy	A_1_R (mice) ( Kochanek et al., 1997)
6-Hz psychomotor seizures test	Acute partial seizures	Low-frequency (6 Hz) stimulation delivered through corneal electrodes	P2X7R (mice) ( Nieoczym et al. 2017)
Maximal electroshock seizure model	Acute generalized tonic-clonic seizures	Electrical stimulation via transcorneal or transauricular electrodes at an intensity sufficient to elicit tonic hind limb extension [50-mA (mice) or 150-mA (rats)]	ADK (rats) ( Ugarkar et al. 2000); P2X7R (mice) (Fischer et al. 2016); P2X7R (mice) ( Nieoczym et al. 2017)
Electrical kindling	Partial epilepsy, partial and secondarily generalized seizures, epileptogenesis	Electrical stimuli via depth electrode in basolateral amygdala, hippocampus, or performant path	P2Rs (rats) (Sun et al. 2018); A_1_R (rats) (Canas et al. 2018)
Seizure-prone mice	Audiogenic seizures	Inbred DBA/2 (D2) mice	ATP release ( Wieraszko and Seyfried, 1989)
Seizure-prone gerbils	From mild hypnotic/ cataleptic to full grand mal seizures	Seizure-sensitive (SS) gerbil	P2X2R and P2X4R (Kang et al. 2003)
Genetic model of absence epilepsy	Absence seizures	Glaxo/Rijswijk (WAG/Rij) rats	Uridine (Kovacs et al. 2013); P2X7R (Dogan et al. 2020)

Abbreviations: ADK, Adenosine kinase; *CCI*, Controlled cortical impact; i.p., intraperitoneal; *KA*, Kainic acid; *PTZ*, Pentylenetetrazol; TLE, Temporal lobe epilepsy.

**Table 2:** Pharmacological approaches to modulate adenosine metabolism and signaling in epilepsy (selected examples).

Receptor subtype / adenosine metabolism	Process	Epilepsy model	Strategy	Outcome	References
**A**_**1**_ **R**	Status epilepticus	Intrahippocampal KA (50 nl of 20 mM solution) in mice (males and females)	A_1_R-KO	Lethal SE in A_1_R-KO mice.	(Fedele et al. 2006)
	Status epilepticus	Theophylline-associated seizures (5, 10, and 25 mg/kg i.p) in rats	DPCPX (0.5 or 5 mg/kg i.p)	A_1_R antagonism led to increased seizure activity and duration.	(Fukuda et al. 2010)
	Epilepsy	i.p. pilocarpine (320 mg/kg) in rats (males)	A_1_R agonist RPia (25 μg/kg/day i.p); A_1_R antagonist DPCPX (50 μg/kg/day i.p) administered for 10 consecutive days, starting from 4 months post-SE	A_1_R agonist reduced seizure rate and hippocampal excitability; while the opposite effect was observed using A_1_R antagonists.	(Amorim et al. 2016)
	Brain injury	Controlled cortical impact (CCI) in mice (males)	A_1_R-KO	Lethal SE in A_1_R-KO mice.	(Kochanek et al. 2006)
	Pharmacoresistant epilepsy	Intrahippocampal injection of KA (200 ng in 50 nl) in mice (males)	A_1_R agonist CCPA (1.5 or 3 mg/ kg i.p)	A_1_R agonism led to suppression of drug resistant seizures in mice.	(Gouder et al. 2003)
	Pharmacoresistant seizures	Human temporal neocortical slices; SLEs e induced by perfusing slices with ACSF containing 8 mM K^+^ and 50 μM bicuculline methiodide	A_1_R agonist SDZ WAG 994 (1 μM); A_1_R antagonist DPCPX (1 μM)	A_1_R agonist completely suppressed SLEs in 73% of slices including slices derived from pharmacoresistant patients; A_1_R antagonism prevented suppression of SLEs.	(Klaft et al. 2016)
**A**_**2A**_ **R**	Epileptogenesis	i.p KA injections (10 mg/kg); Amygdala kindling (1-s train at 50 Hz with pulses of 1 ms and 500 μA) in rats (males)	A_2A_R antagonist, SCH58261 (0.05 mg/kg i.p) administered 30 min before KA injections	A_2A_R antagonism led to suppression of epileptogenesis in amygdala kindled rats; in i.p KA rats, A2AR antagonism prevented seizure-induced neurodegeneration in the hippocampus.	(Canas et al. 2018)
	Epileptogenesis	PTZ kindling (40 – 90 mg/kg, i. p.) and pilocarpine i.p. (350 mg/ kg, i.p.) in mice (sex not specified)	A_2A_R –KO	A_2A_R-KO mice are partially resistant to limbic seizures; A_2A_Rs are involved in excitatory neurotransmission and seizure aggravation.	(El Yacoubi et al. 2009)
**A**_**2B**_ **and A**_**3**_**R**	Epilepsy	Human hippocampal slices	A_2B_R antagonist (MRS1706, 10 nM) or A3R antagonist (MRS1334, 30 nM)	Both A2B and A3R antagonists altered the stability of GABA. Reduced GABA-induced rundown currents in membranes.	(Roseti et al. 2008)
**Adenosine production (CD73)**	Epilepsy	Human TLE and MTLE hippocampus	NT5E measurement through Western blot analysis	Increase in NT5E in tissue from patients with epilepsy	(Barros-Barbosa et al. 2016)
**Adenosine metabolism (ADK)**	Epileptogenesis	Intrahippocampal injection of KA (400 ng in 200 nl) in mice (males)	ADK inhibitor 5-ITU, (1.6 mg/ kg, b.i.d) i.p for 5 days from day 3 to day 8 after SE	5-ITU used transiently after status epilepticus suppress epileptogenesis.	(Sandau et al. 2019)
	Epilepsy	MES-induced seizures in rats (60-Hz current of 150 mA for 0.2 s via corneal electrodes) (males)	5-iodotubercidin, 5-deoxy-5-iodotubercidin and 5-amino-5-deoxyadenosine	ADK inhibitors showed suppression of seizure activity induced by electroshock.	(Ugarkar et al. 2000)
	Epilepsy	Intrahippocampal KA (1 nmol) in transgenic mice (males)	ADK inhibitor, 5ITU (3.1 mg/kg) i.p; Adktm1– /– -Tg(UbiAdk) (ADK overexpressing mice)	ADK overexpressed mice showed spontaneous seizure activity and exacerbation of seizures induced by KA, which was successfully treated by ADK inhibitor 5-ITU.	(Fedele et al. 2005)
	Pharmacoresistant epilepsy	Intrahippocampal injection of KA (200 ng in 50 nl) in mice (males)	ADK inhibitor 5-ITU, (3.1 mg/ kg), i.p	ADK inhibitors suppressed seizure activity including pharmacoresistant seizures.	(Gouder et al. 2004)

Abbreviations: *ADK,* Adenosine kinase; *ACSF*, Artificial cerebrospinal fluid; *CCPA*, 2-chlorN6cyclopentyladenosine; *DPCPX*, 8-Cyclopentyl-1,3-dipropylxanthine; *GABA*, Gama amino butyric acid; *i.p.*, intraperitoneal; *KA*, Kainic acid; *KO*, knock out; MES, Maximal electric shock; *MRS1334*, 1,4-Dihydro-2-methyl-6-phenyl-4-(phe-nylethynyl)-3,5-pyridinedicarboxylic acid 3-ethyl-5-[(3-nitrophenyl)methyl] ester; *MRS1706*, N-(4-Acetylphenyl)-2-[4-(2,3,6,7-tetrahydro-2,6-dioxo-1,3-dipropyl-1H-purin-8-yl)phenoxy]acetamide; *MTLE*, Mesial temporal lobe epilepsy; *NT5E*, Ecto-5’-nucleotidase; *PTZ*, Pentylenetetrazol; *5ITU*, 5-indotubercidin; *SE*, Status epilepticus; *SLE*, Seizure-like events; *TLE*, Temporal lobe epilepsy.

**Table 3:** Impact of P2 receptor-targeting during acute seizures, epileptogenesis and epilepsy (selected examples).

** Receptor subtype**	** Process**	** Epilepsy model**	** Strategy**	** Outcome**	** References**
**Ionotropic P2XRs**					
**P2X7R**	Status epilepticus	i.p. KA (25 mg/kg), i.p. picrotoxin (5 mg/kg), i.p. pilocarpine (150, 175, 200, 225, or 250 mg/kg) in mice (males)	i.c.v. OxATP (5 mM), A-438079 (10 μM), A740003 (10 μM) delivered over 1 week via osmotic mini-pump before seizure induction; P2X7R KO mice	Increased seizure susceptibility via P2X7R antagonism; P2X7R antagonisms did not affect seizures in KA and picrotoxin model.	(Kim and Kang, 2011)
	Status epilepticus	i.p. pilocarpine (380 mg/kg) in rats (males)	i.c.v. B_Z_ATP (5 mM), OxATP (5 mM), A-740003 (5 mM) and A-438079 (10 μM) via osmotic mini-pump	Reduced neurodegeneration via BzATP; increased neurodegeneration, reduced astroglial death and reduced infiltration of neutrophils mediated via P2X7R antagonism.	(Kim et al. 2009), (Kim et al. 2011), (Kim et al. 2010)
	Status epilepticus	i.a. KA (3 μg/2 μl) in mice (males)	P2X7R agonists: i.c.v. BZATP (0.1 nmol); P2X7R antagonists: i.c.v. A438079 (1.75 nmol), i.c.v. BBG (1 pmol); i.c.v. P2X7R antibody (APR-008, 0.7 mg/mL); P2X7R KO mice	P2X7R agonist-increased seizure severity; P2X7R antagonists / P2X7R-targeting antibodies / P2X7R KO leads to seizure reduction and neuroprotection in hippocampus and cortex.	(Engel et al. 2012), ( Jimenez-Pacheco et al. 2013)
	Status epilepticus	i.m. coriaria lactone (40 mg/kg) in rats (males)	Pre-treatment with P2X7R antagonists BBG (1, 5, 10 μg; i.c. v.), A438079 (10 μM, ic.v.) and A740003 (10 μM, i.c.v.)	P2X7R antagonism reduced inflammation, neuronal damage, astrogliosis and microgliosis, seizures and improved cognitive function.	(Huang et al. 2017)
	**Acute seizures (focal, generalized and generalized tonic-clonic)**	Timed i.v. PTZ infusion test (1% PTZ 2 ml/min) in mice (males); MES-T; 6 Hz electroshock-induced seizures (0.2 ms square pulse at 6 Hz for 3 s) in mice (males)	i.p. BBG 150 mg/kg for i.v. PTZ and MES-T test and i.p. BBG 50 mg/kg for 6 Hz test	Reduced seizures during 6 Hz test (focal seizure) via BBG; no significant anticonvulsive effects of BBG in i.v. PTZ and MES-T test (generalized and generalized tonic-clonic seizures).	(Nieoczym et al. 2017)
	Acute seizures (absence seizures)	WAG/Rij rats (males) (inbred strain of rats with genetic absence epilepsy)	i.c.v. BZATP (50 μg and 100 μg); i.c.v. A-438079 (20 μg and 40 μg)	No effects of P2X7R agonists or antagonists on spike-wave discharges (SWDs)	(Dogan et al. 2020)
	Acute seizures / Epileptogenesis	MES-T (inusoidal pulses 4–14 mA, 50 Hz, 0.2 s duration) and s.c. PTZ-T (87 mg/kg) in mice (males); i.p. PTZ kindling (35 mg/ kg) in rats (males) for 25 days	JNJ-47965567 (15 or 30 mg/ kg),AFC-5128 (25 or 50 mg/ kg), BBG (50 mg/kg), transhinone (30 mg/kg), all drugs injected i.p.	No effects on acute seizures alone; reduced seizure severity in combination with carbamazepine; reduced kindling development and glial activation.	(Fischer et al. 2016)
	Epileptogenesis	i.p. PTZ kindling (30 mg/kg) every second day for 27 days in rats (sex not specified)	BBG (15 and 30 mg/kg, i.p.) 30 min before PTZ injection	Reduced seizure score and improved motor performance and cognitive deficits.	(Soni et al. 2015)
	Epileptogenesis	i.a. KA (3 μg/2 μl)-induced epilepsy in mice (males)	i.c.v. injection of antagomir-22 24 h before induction of SE	Increased P2X7R; increased seizure frequency during epilepsy, increased neuroinflammation.	(Jimenez-Mateos et al. 2015)
	Epileptogenesis	i.p. pilocarpine (370 mg/kg,i.p.) in rats (males)	P2X7R antagonists AZ10606120 (3 μg/2 μl, i.c.v.) post-SE / BBG (50 mg/kg, i.p.) 1 injection per day for 4 days post-SE	Neuroprotection mediated via P2X7R antagonism post-SE; P2X7R antagonisms increased seizure number and seizure severity during epilepsy.	(Rozmer et al. 2017)
	Epileptogenesis	i.p. pilocarpine (370 mg/kg) in rats (males)	P2X7R-targeting siRNA (i.c.v.) 6 h post-SE	P2X7R antagonisms mediated neuroprotection in hippocampus, reduced edema, reduced mortality following SE, delayed seizure onset and seizure numbers during chronic epilepsy.	(Amorim et al. 2017)
	Epilepsy	Multiple low-dose i.p. KA-induced epilepsy in rats (males) (total KA = 22.2 ± 2.02 mg/kg)	JNJ-47965567 during 1 week via osmotic mini-pump (0.6 g/ kg/2 ml)	Decreased seizure severity without changes in total number of seizures, no change in inflammation.	(Amhaoul et al. 2016)
	Epilepsy	i.a. KA (3 μg/2 μl)-induced-epilepsy in mice (males)	JNJ-47965567 (30 mg/kg, i.p.) twice daily for 5 days during epilepsy	Reduced seizure frequency during treatment and during washout-period; decreased inflammation (astrogliosis and microgliosis).	(Jimenez-Pacheco et al. 2016)
**P2X3R**	Epileptogenesis	i.p. PTZ (30 mg/kg) administered on alternate days for a maximum of up to 35 days to rats (males)	P2X3R antagonist i.c.v. delivery of P2X3 antagonist NF110 in 3 different doses (20 nM, 40 nM, and 60 nM) from day 1 until the end of the study (day 42)	P2X3R antagonisms improved impaired behavior, learning, memory, locomotion, motor activity, discrimination ability, neuronal damage, hippocampal inflammation, oxidative stress, and mitochondrial dysfunction	(Xia et al. 2018)
**P2X4R**	Status epilepticus	i.p. KA (8–22 mg/kg) in mice (sex not specified)	P2X4R KO mice	Impaired microglial function; neuroprotection in hippocampus.	(Ulmann et al. 2013)
**Metabotropic P2Y receptors**					
**P2Y**_**1**_ **R**	Status epilepticus	i.p. KA (10 mg/kg) in rats (males); intrahippocampal injection of quinolinic acid (1 μl at the rate of 0.2 μL/min) in rats (males)	One single injection of MRS2500 (nmol, i.c.v.)	P2Y_1_R antagonist-mediated neuroprotection; no impact on seizure severity.	(Simoes et al. 2018)
	Status epilepticus / Epileptogenesis / Epilepsy	i.a. KA (3 μg/2 μl) in mice; i.p. pilocarpine (340 mg/kg) in mice (males)	P2Y_1_R antagonist MRS2500 (1 nmol, i.c.v.) before and during SE, post-SE and during chronic epilepsy once daily for 5 days, P2Y_1_R KO mice; P2Y_1_R agonist MRS2365 (0.3 and 1 nmol, i.c. v.) before and during SE	*P2Y*_*1*_R *KO mice:* Increase in seizure severity during SE and neurodegeneration. *Pre-treatment:* P2Y_1_R agonist reduces seizure severity during SE and protects brain from damage; P2Y_1_R antagonism increases seizure severity and brain damage during SE. *Treatment during SE:* P2Y_1_R agonist increases seizure severity during SE; P2Y_1_R antagonism decreases seizure severity and brain damage (hippocampus and cortex). *P2Y*_*1*_*R antagonism post-SE:* Delay in epilepsy development. *P2Y*_*1*_*R antagonism during epilepsy:* Suppression of epileptic seizures.	(Alves et al. 2019); ( Alves et al. 2019)
	**P2Y**_**12**_ **R**	Status epilepticus	i.p. KA (18–22 mg/kg) or i.c.v. KA (0.12–0.18 μg)and KA in mice (males and females)	P2Y_12_R KO mice Increased seizure phenotype; reduced hippocampal microglial processes.	(Eyo et al. 2014)
**Broad-spectrum targeting**					
	Status epilepticus	i.a. KA (3 μg/2 μl) in mice (males)	ADP and UTP (P2Y agonists) (9 nmol i.c.v.)	ADP exacerbates seizure severity; UTP reduces seizure severity and neuronal death.	(Alves, Gomez-Villafuertes et al. 2017)
	Epilepsy	Amygdala kindling in rats (males) (1 s, monophasic square-wave pulses)	Reactive Blue (P2YR antagonists, 20 μg) and PPADS (P2 antagonist, 10, 20, 30 μg) once daily i.c.v.	Reduced seizure severity.	(Sun et al. 2018)

Abbreviations: *ADP*, Adenosine diphosphate*; BBG*, brilliant blue G; *BzATP*, 2’(3’)-O-(4-Benzoylbenzoyl)adenosine-5’-triphosphate; *i.a*., intraamygdala; *i.m.* intra-muscular; *i.p.*, intraperitoneal; *i.c.v.*, intracerebroventricular; *i.v.*, intravenous; *KA*, Kainic acid; *KO*, Knock out; *MES-T*, Maximal electroshock seizure threshold test; *PPADS*, Pyridoxalphosphate-6-azophenyl-2’,4’-disulfonic; *PTZ-T*, Pentylenetetrazol seizure threshold test; *SE*, Status epilepticus; *UDP*, Uridine diphosphate; *UTP*, Uridine triphosphate.

**Table 4:** Table summarizing substrate specificity, Kms and tissue distribution of ENTs.

ENTs	Km values (Adenosine)	Substrate specificity	Tissue distribution	References
ENT1	40 μM	Adenosine, purine and pyrimidines nucleosides	Ubiquitous. Predominant in adrenal gland, kidneys, stomach, small intestine. Also present in colon and ovaries.	(Griffith and Jarvis, 1996; Kong et al. 2004; Inoue, 2017)
ENT2	100 μM	Adenosine, purine and pyrimidines nucleosides, nucleobases	Ubiquitous. Highest in skeletal muscles. Also abundant in brain, neural tissues, heart, placenta, GI tract	(Griffith and Jarvis, 1996; Kong et al. 2004)
ENT3	2 mM	Adenosine, purine and pyrimidines nucleosides	Intracellular membranes, cerebral cortex, ovary, adrenal gland, and testis.	(Hyde et al. 2001; Baldwin et al. 2005)
ENT4	0.78 mM	Adenosine, serotonin	Abundant in heart, especially vascular endothelial cells.	(Baldwin et al. 2004; Barnes et al. 2006)

**Abbreviations:** *ENT*, Equilibrative nucleotide transporter; *GI*, Gastrointestinal.

## Abbreviations

5-ITU: 5-Iodotubercidin
ADA: Adenosine deaminase
ADK: Adenosine kinase
AR: Adenosine receptor
ASD: Anti-seizure drug
BBB: Blood-brain barrier
BBG: Brilliant Blue G
BZATP: 2’,3’-O-(4-benzoyl-benzoyl)ATP
cAMP: cyclic adenosine monophosphate
CD39: Ectonucleoside triphosphate diphosphohydrolase
CD73: Ecto-5’-nucleotidase
CNT: Concentrative nucleoside transporters
DNMT: DNA methyltransferase
EEG: Electroencephalogram
EGFP: Enhanced green fluorescent protein
ENT: Equilibrative nucleoside transporters
GABA: γ-aminobutyric acid
HMSC: Human mesenchymal stem cells
HCY: Homocysteine
IL-1β: Interleukin-1β
KA: Kainic acid
KD: Ketogenic diet
KO: knock-out
MAD: Modified Atkins diet
MTLE: Mesial temporal lobe epilepsy
NMDA: N-methyl-D-aspartate receptor
NLRP3: NACHT, LRR and PYD domains-containing protein 3
NT5E: ecto-5’-nucleotidase
P2XR: P2X receptor
P2YR: P2Y receptor
PTE: post-traumatic epilepsy
PTZ: Pentylenetetrazole
SAH: S-adenosyl-L-homocysteine
SAM: S-adenosylmethionine
Sp1: Specificity protein 1
SUDEP: Sudden unexpected death in epilepsy
TBI: Traumatic brain injury
TLE: Temporal lobe epilepsy
UDP: Uridine diphosphate
UTP: Uridine triphosphate.
